# Cleavage kinetics of human mitochondrial RNase P and contribution of its non-nuclease subunits

**DOI:** 10.1093/nar/gkad713

**Published:** 2023-10-02

**Authors:** Elisa Vilardo, Ursula Toth, Enxhi Hazisllari, Roland K Hartmann, Walter Rossmanith

**Affiliations:** Center for Anatomy & Cell Biology, Medical University of Vienna, 1090 Vienna, Austria; Center for Anatomy & Cell Biology, Medical University of Vienna, 1090 Vienna, Austria; Center for Anatomy & Cell Biology, Medical University of Vienna, 1090 Vienna, Austria; Institute of Pharmaceutical Chemistry, Philipps-University Marburg, 35037 Marburg, Germany; Center for Anatomy & Cell Biology, Medical University of Vienna, 1090 Vienna, Austria

## Abstract

RNase P is the endonuclease responsible for the 5′ processing of precursor tRNAs (pre-tRNAs). Unlike the single-subunit protein-only RNase P (PRORP) found in plants or protists, human mitochondrial RNase P is a multi-enzyme assembly that in addition to the homologous PRORP subunit comprises a methyltransferase (TRMT10C) and a dehydrogenase (SDR5C1) subunit; these proteins, but not their enzymatic activities, are required for efficient pre-tRNA cleavage. Here we report a kinetic analysis of the cleavage reaction by human PRORP and its interplay with TRMT10C-SDR5C1 including 12 different mitochondrial pre-tRNAs. Surprisingly, we found that PRORP alone binds pre-tRNAs with nanomolar affinity and can even cleave some of them at reduced efficiency without the other subunits. Thus, the ancient binding mode, involving the tRNA elbow and PRORP’s PPR domain, appears basically retained by human PRORP, and its metallonuclease domain is in principle correctly folded and functional. Our findings support a model according to which the main function of TRMT10C-SDR5C1 is to direct PRORP’s nuclease domain to the cleavage site, thereby increasing the rate and accuracy of cleavage. This functional dependence of human PRORP on an extra tRNA-binding protein complex likely reflects an evolutionary adaptation to the erosion of canonical structural features in mitochondrial tRNAs.

## Graphical Abstract

**Graphical Abstract:**
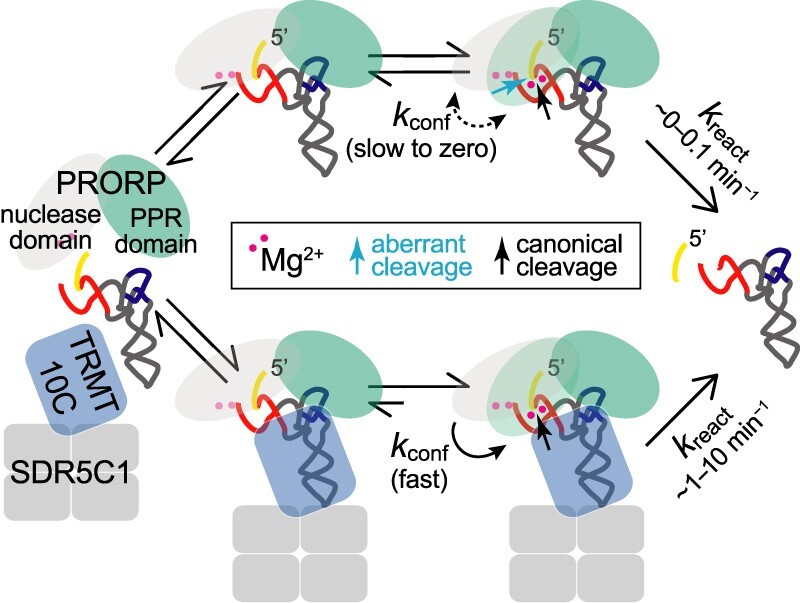

## INTRODUCTION

In human mitochondria, RNAs are transcribed as long polycistronic precursors, from which mRNAs, rRNAs and tRNAs have to be released by endonucleases before further maturation to their final functional forms. Due to the characteristic interspersed arrangement of the tRNA sequences, the two enzymes RNase P and RNase Z, responsible for 5′- and 3′-end processing of tRNAs, concomitantly generate the ends of the two rRNAs and most mRNAs, and thereby account for almost the entire primary processing of mitochondrial transcripts (1–4). While mitochondrial RNase Z is derived from the same gene (*ELAC2*) as its nuclear isoform (1,5,6), mitochondrial RNase P (mtRNase P) is different from and unrelated to nuclear RNase P (1,7–9). Remarkably, mtRNase P is also a multi-enzyme assembly that appears exceptional, even within the diverse RNase P enzyme family (10). In the eukaryal domain, the RNase P family encompasses ribonucleoproteins of various levels of complexity, as well as a class of comparatively ‘simple’, monomeric 60-kDa protein enzymes called proteinaceous RNase P (PRORP) (10,11). PRORP proteins function as RNase P in the nucleus, mitochondria, and/or plastids of land plants, algae, trypanosomes, and probably several other eukaryal groups (12–18). Also, human mtRNase P comprises a PRORP protein (also known as MRPP3) as endonuclease, but it requires two additional essential protein subunits that have further functions, unrelated to their role as RNase P subunits (9,19): short-chain dehydrogenase/reductase family 5C member 1 (SDR5C1, also MRPP2 or HSD17B10) catalyzes the penultimate step in the β-oxidation of short branched-chain fatty and amino acids (20), and tRNA methyltransferase 10C (TRMT10C, also MRPP1) forms a stable subcomplex with SDR5C1 that constitutes the methyltransferase responsible for *N*^1^-methylation of purines at position 9 of mitochondrial tRNAs (19).

We previously showed that SDR5C1 is not directly involved in tRNA binding and neither its dehydrogenase activity nor an intact binding site for its NAD^+^/NADH cofactor are required for the methyltransferase or endonucleolytic activity of the mtRNase P complex, suggesting a scaffolding role for SDR5C1 (10,19,21). Similarly, the methyltransferase activity of TRMT10C is dispensable for cleavage by PRORP, supporting a tRNA-binding and structural, rather than enzymatic role of the TRMT10C-SDR5C1 complex in promoting the cleavage reaction (19). Recently, the cryo-electron microscopy (cryo-EM) structure of human mtRNase P bound to precursor tRNA (pre-tRNA) was reported, showing that TRMT10C encases part of the tRNA, and forms several specific and nonspecific interactions with all four subdomains of the tRNA structure (22). TRMT10C is anchored to the SDR5C1 tetramer via a central loop and helix connecting its two tRNA-interacting domains, while SDR5C1 itself appears to have limited contact with the anticodon loop of the tRNA. An arch-like shaped PRORP binds on top of the pre-tRNA–TRMT10C–SDR5C1 complex through interactions with both, the pre-tRNA and TRMT10C; its nuclease domain contacts the pre-tRNA around the cleavage site and the methyltransferase domain of TRMT10C, and on the other end, its pentatricopeptide repeat (PPR) domain interacts with the elbow of the tRNA structure and the N-terminal domain of TRMT10C. Active-site organization and the overall geometry of the PRORP-tRNA interaction appear similar to that of (single-subunit) PRORPs from *Arabidopsis thaliana* based on avaiable crystal structures or models of PRORP-tRNA complexes (23–26), yet the specific molecular interactions between the PPR domain and the tRNA may vary (22,27).

The structure of the mtRNase P-pre-tRNA complex confirmed the originally proposed role of the TRMT10C–SDR5C1 complex as an accessory factor in the recognition of pre-tRNAs (9,10,22). However, a suggested role in remolding the disordered catalytic domain observed in the crystal structures of PRORP fragments, to enable the coordination of Mg^2+^ in the nuclease's active site, appears not entirely consistent with the reported structural and enzymatic data (22,28,29). Thus, the actual mechanisms behind the interplay between TRMT10C–SDR5C1 and PRORP leading to pre-tRNA cleavage had so far remained unclear.

Here we report a detailed analysis of tRNA 5′ maturation by human mtRNase P using a comprehensive and representative set of mitochondrial pre-tRNA substrates. We investigated the contribution of the individual mtRNase P subunits to 5′-end processing, their pre-tRNA-binding kinetics, and the cleavage process itself under a variety of kinetic regimens. We also examined previously raised hypotheses about the role(s) of TRMT10C–SDR5C1. Altogether our results reveal new insights into the mechanism and conformational dynamics of an exceptionally multifaceted member of the diverse RNase P enzyme family.

## MATERIALS AND METHODS

### Expression and purification of recombinant proteins

C-terminally His_6_-tagged human PRORP, C-terminally myc-His_6_-tagged TRMT10C, N-terminally His_6_-tagged SDR5C1, N-terminally His_6_-tagged *Saccharomyces cerevisiae* Trm10p, and C-terminally His_6_-tagged *Arabidopsis thaliana* PRORP3 were prepared as previously described (19,30). The TRMT10C-SDR5C1 complex was essentially prepared as previously described (19), with the imidazole concentration during the wash step increased to 200 mM.

Substitutions D479N and D499N were introduced into the PRORP expression plasmid by site-directed mutagenesis using the QuikChange protocol (Agilent Technologies). The two variants were expressed and purified like the wild type protein (19).

ELAC2, starting with amino acid 16, was cloned into the *Nde*I/*Xho*I sites of pET-21b(+) and, including the stop codon, into the same sites of pET-28b(+) (Novagen); thereby the recombinant proteins include either a C-terminal or an N-terminal His_6_ tag. C- and N-terminally His_6_-tagged ELAC2 (indicated as ELAC2-His and His-ELAC2, respectively) were expressed in *Escherichia coli* Rosetta2(DE3) and purified on HisTrap HP columns (Cytiva) essentially as previously described for SDR5C1 (19): proteins were loaded and washed with 50 mM imidazole, then washed with 1 M NaCl, followed by 300 mM imidazole, and eluted with 500 mM imidazole (all in buffer A).

The purity of the recombinant proteins and the stoichiometry of the TRMT10C-SDR5C1 complex were assessed by SDS-PAGE and Coomassie brilliant blue staining, and found to be >95% and consistent with a 2:4 ratio, respectively. All protein concentrations were quantitated relative to bovine serum albumin standards by SDS-PAGE, Coomassie brilliant blue staining, and image analysis using ImageQuant TL 8 (Cytiva); although more laborious, this gel-based, relative quantitation was found to be more reliable than standard UV_280_ measurements, as it is not affected by impurities or buffer components (e.g. imidazole), and avoids calculations based on insensitive extinction coefficients (e.g. SDR5C1 lacks tryptophanes). In the case of the purified TRMT10C–SDR5C1 complex, the indicated concentrations refer to the concentration of TRMT10C monomers as the presumable active tRNA-binding unit.

### Precursor tRNA substrates

The pre-tRNAs used for RNase P activity assays were human mitochondrial tRNAs with short stretches of their natural 5′- and 3′-flanking sequences. The plasmid constructs for *in vitro* transcription of pre-tRNA^Tyr^, pre-tRNA^Ile^ and pre-tRNA^His^ were previously described (9,19). Similarly, plasmid constructs for *in vitro* transcription of the other pre-tRNAs were generated by PCR cloning of the following regions of the human mitochondrial genome (nucleotides according to the revised Cambridge reference sequence, whereby genes encoded by the L-strand are indicated by forward, those by the H-strand by reverse numbering; refs. 31,32): 5691–5567 for pre-tRNA^Ala^, 5891–5738 for pre-tRNA^Cys^, 4434–4312 for pre-tRNA^Gln^, 14836–14639 for pre-tRNA^Glu^, 8254–8375 for pre-tRNA^Lys^, 4368–4503 for pre-tRNA^Met^, 16060–15926 for pre-tRNA^Pro^, 7521–7441 for pre-tRNA^Ser(UCN)^ and 1571–1702 for pre-tRNA^Val^. Their *in vitro* transcripts start in most cases with a short stretch of polylinker sequence preceding the natural 5′-flanking sequence of the respective tRNA (see Supplementary Figure S1A for the complete sequences of all pre-tRNA transcripts and Supplementary Figure S2 for their secondary structure). The template for the RNase P model substrate *Thermus thermophilus* pre-tRNA^Gly^ was previously described (30,33).

Pre-tRNAs used for RNase Z activity assays were 5′-mature human mitochondrial tRNAs with a short stretch of 3′-flanking sequence only (Supplementary Figures S1B and S2). For pre-tRNA^His^ (12138–12223), pre-tRNA^Leu(UUR)^ (3230–3340) and pre-tRNA^Ser(UCN)^ (7514–7426), transcription templates were derived by *in vitro* mutagenesis of plasmids encoding tRNAs with 5′- and 3′-flanking sequences; thereby all sequences between the T7/T3 polymerase promoter initiation site and the 5′ end of the tRNA were deleted to start *in vitro* transcription directly with the G1 of the tRNA. In the templates for pre-tRNA^Ala^ (5655–5567) and pre-tRNA^Ile^ (4263–4351), the tRNA 5′ end was fused to a hammerhead ribozyme to release the 5′ end of the tRNA after *in vitro* transcription. The plasmid construct for pre-tRNA^Ala^ was assembled by overlap extension PCR and cloned as previously described (19), the one for pre-tRNA^Ile^ was a kind gift from Louis Levinger (34).

*In vitro* transcription, 5′-end labeling with [γ-^32^P]ATP and purification of pre-tRNAs were carried out as previously described (7,35). The concentration of unlabeled pre-tRNAs was determined by UV spectrophotometry, the one of labeled pre-tRNAs, because of their lower concentration, by a fluorimetric assay (RiboGreen RNA quantitation reagent; Molecular Probes) using standard curves generated by serial dilution of unlabeled pre-tRNA.

### RNase P and RNase Z cleavage assays

Qualitative RNase P cleavage assays were performed at 21°C as previously described (9,21; the low reaction temperature was originally found to be beneficial for enzyme stability of partially purified native preparations of mtRNase P). Quantitative kinetic analyses, however, were performed at 30°C; at this temperature the reaction was slow enough to allow manual sampling of time points at otherwise unchanged reaction conditions, and besides, it corresponds to the temperature typically used in studies of mitochondrial bioenergetics (36–40). The previously described mtRNase P reaction buffer (50 mM Tris·Cl pH 8, 20 mM NaCl, 2 mM DTT, 20 μg/ml BSA, 0.5 units/μl recombinant RNase inhibitor; ref. 21) was supplemented with either 3 or 4.5 mM Mg^2+^ here, as found optimal for the cleavage of the respective pre-tRNA and indicated in the figure legends. The entire experimental procedure was detailed previously (30) and the differences are described in the following.

In single-turnover kinetic experiments (final substrate concentration 0.2 nM), labeled pre-tRNAs and PRORP, each at twice the final concentration, were pre-incubated separately in reaction buffer at 30°C, and reactions started by combining equal volumes of the two. To analyze PRORP cleavage in the presence of the TRMT10C-SDR5C1 complex (mtRNase P holoenzyme), the latter was added to the substrate at the preincubation step to 400 nM (i.e. 200 nM final concentration, which was verified to be saturating for all tested substrates in pilot experiments). A similar regimen was followed in multiple-turnover kinetic experiments, but the pre-tRNA substrates were pre-incubated with a fourfold molar excess of TRMT10C–SDR5C1 (or at least 200 nM final concentration in the case of the lower substrate concentrations); the different concentrations of unlabeled substrate in multiple-turnover experiments were spiked with labeled substrate at 1 or 2 nM. The concentration of PRORP in multiple-turnover kinetic experiments was 1 nM. Sampling and all further processing of withdrawn aliquots were carried out as previously described (30).

Aliquots of single-turnover reactions with the mtRNase P holoenzyme (PRORP plus TRMT10C-SDR5C1) were sampled throughout the reaction and pseudo first-order rate constants of cleavage (*k*_obs_) were calculated by nonlinear regression analysis fitting the data to the equation for a single exponential: *f*_cleaved_ = *f*_endpoint_ × (1 − e^−(*k*_obs_) ×^*^t^*), where *f*_cleaved_ = fraction of substrate cleaved, *t* = time, *f*_endpoint_ = maximum cleavable substrate (Prism, GraphPad Software; Supplementary Figure S3 shows the gel and the analysis of a representative experiment). Single-turnover reactions with PRORP alone were considerably slower and, particularly at lower enzyme concentrations, did not reach meaningful endpoints within reasonable incubation times. Therefore, aliquots were sampled throughout the initial, largely linear phase of the reactions, only, and linear regression analysis was used to approximate the pseudo first-order rate constants (*k*_obs*_) of cleavage (Supplementary Figure S4 shows the gel and the analysis of a representative experiment). Similarly, aliquots of multiple-turnover reactions were sampled throughout the initial, largely linear phase of the reactions, and linear regression analysis was used to approximate the initial velocity (*v*) of cleavage (Supplementary Figure S5 shows the gel and the analysis of a representative experiment).

For the analysis of single-turnover kinetics, *k*_obs_ or *k*_obs*_ values from at least six different enzyme concentrations, from at least four replicate experiments each, were plotted as mean ± standard error of the mean (SEM) against the enzyme concentration; the maximal rate constant (*k*_react_) and the enzyme concentration at which the half-maximal rate constant is achieved (*K*_M(sto)_) were calculated by nonlinear regression, fitting the individual *k*_obs_ or *k*_obs*_ values to a ‘Michaelis-Menten-like’ enzyme kinetics model: *k*_obs(*)_ = *k*_react_ × [PRORP]/(*K*_M(sto)_ + [PRORP]). For the analysis of multiple-turnover kinetics, *v* values from at least seven different substrate concentrations, from at least four replicate experiments each, were plotted as mean ± SEM against the substrate concentration; the turnover number (*k*_cat_) and the Michaelis–Menten constant (*K*_M_) were calculated by nonlinear regression, fitting the individual *v* values to a Michaelis-Menten enzyme kinetics model: *v* = [PRORP] × *k*_cat_ × [pre-tRNA] / (*K*_M_ + [pre-tRNA]). The results are reported as best-fit values ± curve-fit standard error (Prism, GraphPad Software).

Pulse-chase reactions were essentially carried out as previously outlined (41). In brief, single-turnover kinetic reactions with 50 nM PRORP and 200 nM TRMT10C-SDR5C1 were set up as described above. After the indicated time the reactions were chased either by the addition of unlabeled pre-tRNA to 1 μM (increase in volume < 5%) or by 200-fold dilution with reaction buffer. Samples from the diluted reactions were precipitated with ethanol/ammonium acetate/glycogen before dissolving them in the gel loading buffer. Further processing and analysis were carried out as described above.

To determine the optimal Mg^2+^ concentration for pre-tRNA cleavage, enzyme kinetics were carried out at Mg^2+^ concentrations ranging from 0.5 or 1.5 up to 9 or 12 mM, with 200 nM PRORP alone, or with 20 nM PRORP plus 200 nM TRMT10C–SDR5C1 complex; *k*_obs_ values were derived from at least three replicate experiments.

RNase Z cleavage and kinetics were performed at 30°C in a buffer composed like the mtRNase P reaction buffer, but with pH 7.4 (because ELAC2 was unstable at pH 8) and 1 mM Mg^2+^. Assays and analyses of single-turnover kinetics were carried out as described above for the mtRNase P holoenzyme.

### RNA binding studies

The binding of the subunits of mtRNase P to different mitochondrial pre-tRNAs was measured by bio-layer interferometry on a BLItz instrument (Pall FortéBio). The method allows the label-free kinetic characterization of macromolecular interactions in real time. One of the binding partners (the pre-tRNA in our case) is immobilized on a sensor tip, through which a beam of white light is projected, and the interference pattern of light reflected at the surface and an internal reference layer is recorded. The binding of interacting macromolecules (here, the subunits of mtRNase P) changes the interference pattern, and the shift, expressed in nanometers, is a measure of binding. By following the association and dissociation of the proteins, the kinetic rate constants *k*_on_ and *k*_off_ of the RNA-protein interaction can be determined, and the *K*_D_ (*k*_off_/*k*_on_) derived thereof. Measurements at different concentrations improve the accuracy of the analysis, but are not a precondition, nor do concentrations have to span the range below and above *K*_D_ like in equilibrium binding approaches.

For immobilization on streptavidin-coated sensors, pre-tRNAs were biotinylated at either their 5′ or 3′ end. Briefly, *in vitro* transcribed pre-tRNA^Ala^ and pre-tRNA^His^ (see previous section) were ligated to a 5′-biotinylated DNA oligonucleotide (bio-TGTGGTTGACGTGC) using a complementary bridging oligonucleotide (splint) and T4 DNA ligase (42). For pre-tRNA^Lys^ and pre-tRNA^Val^, the regions corresponding to nucleotides 9–73 plus 3′-flanking sequence (8303–8375 and 1610–1702 of the mitochondrial genome, respectively) were amplified by PCR, fused at their 5′ end to a hammerhead ribozyme-coding cassette by overlap-extension PCR, and cloned into the plasmid pGEM-1 (Promega) as previously described (19). After hammerhead release, the *in vitro* transcribed tRNA fragments were completed by ‘splint ligation’ to a synthetic RNA corresponding to the first 8 nucleotides of the respective tRNA plus 15 nucleotides of the natural 5′-flanking sequence (19). Pre-tRNA^Lys^ and pre-tRNA^Val^ were 3′ biotinylated with pCp-biotin (750 μM; Jena Bioscience) and T4 RNA ligase (2 units/μl), at 16°C overnight in T4 RNA ligation buffer (10 mM Tris·Cl pH 7.8, 10 mM MgCl_2_, 10 mM DTT, 1 mM ATP). Gel-purified pre-tRNAs were immobilized on streptavidin-coated sensors, and protein binding was assayed upon incubation with different concentrations in the range of 25–1000 nM (interference signals were too weak below 50 nM of PRORP or TRMT10C, and below 25 nM of TRMT10C-SDR5C1). The number of different concentrations used for PRORP, TRMT10C and TRMT10C–SDR5C1, respectively, were 6, 4 and 4 for tRNA^Ala^; 5, 4 and 4 for tRNA^His^; 5, 3 and 5 for tRNA^Lys^; and 5, 4 and 4 for tRNA^Val^. All steps were performed at controlled room temperature set to 21°C (the instrument itself does not allow temperature control) in the following binding buffer: 50 mM Tris·Cl pH 8, 20 mM NaCl, 2 mM DTT, 20 μg/ml BSA, 0.5 units/μl recombinant RNase inhibitor, 0.05% Tween-20 (to minimize unspecific binding to the sensor), with or without 4.5 mM MgCl_2_ (Mg^2+^ was included in the analysis of tRNA^Lys^, but excluded in the case of the other pre-tRNAs to prevent cleavage by PRORP; in pilot experiments, Mg^2+^ was found to not significantly affect binding by TRMT10C or PRORP). Association and dissociation were recorded for 3–5 and 5–30 min, respectively (depending on the respective pre-tRNA·protein interaction studied), and the binding data were analyzed with the BLItz Pro software (Pall FortéBio) applying step correction and global fitting; results are reported as best-fit values ± curve-fit standard error.

### Iron-mediated cleavage of PRORP

For Fe(II)-mediated hydroxyl radical cleavage (43), 7.2 μM PRORP was incubated with 200 μM (NH_4_)_2_Fe(SO_4_)_2_, 10 mM DTT, and 10 mM ascorbic acid, in 50 mM PIPES-K pH 6.7 buffer. TRMT10C-SDR5C1 complex and/or pre-tRNA^Ile^ (see section above) were added to a final concentration of 7.8 and 7.5 μM, respectively. Reactions were incubated at 20°C for 60 min, stopped by addition of 5 × Laemmli buffer, and analyzed by 12–20%-gradient SDS-PAGE and Coomassie brilliant blue staining. The identity of the C-terminal fragments produced by Fe(II)-mediated cleavage was confirmed by western blotting using an anti-His-tag antibody.

## RESULTS

### PRORP alone is able to cleave pre-tRNAs *in vitro*

**Figure 1.**
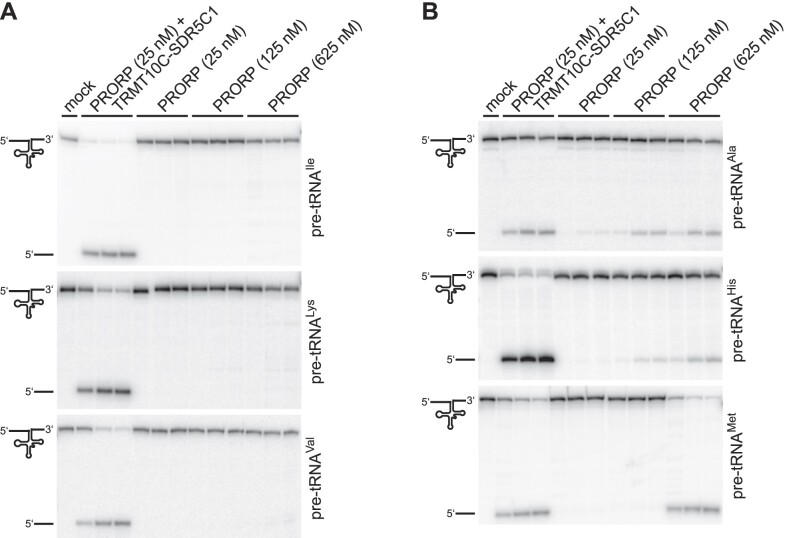
Human PRORP has RNase P activity without TRMT10C-SDR5C1. The RNase P activity of PRORP was tested on model substrates of the six indicated human mitochondrial pre-tRNAs. Aliquots were withdrawn from the reactions after 3, 30 and 60 min for pre-tRNA^Ala^ and pre-tRNA^Val^, and 10, 30 and 60 min for pre-tRNA^His^, pre-tRNA^Ile^, pre-tRNA^Lys^ and pre-tRNA^Met^; cleavage products were separated by gel electrophoresis and visualized by phosphorimaging. The final concentration of the TRMT10C–SDR5C1 complex was 250 nM. No enzyme was added to the ‘mock’ reaction, which was incubated for 60 min. Due to 5′-end labeling, only the full-length pre-tRNA and the released 5′ leader are visible. (**A**) Examples of mitochondrial pre-tRNAs whose 5′-end processing requires both, PRORP and the TRMT10C–SDR5C1 complex, or (**B**) on which PRORP alone showed RNase P activity *in vitro*.

With the identification of the subunits of human mtRNase P, we originally found all three proteins to be required for the reconstitution of the pre-tRNA-cleavage activity (9). However, the recombinant forms of various PRORP homologs later identified in plants or protists were found to have RNase P activity on their own, without the involvement of additional proteins (12–17). The architecture of human PRORP moreover shows all the defining features generally found in PRORP homologs (18), i.e. a characteristic C-terminal NYN metallonuclease domain, an N-terminal α-super helical domain containing pentatricopeptide repeat (PPR) motifs, and a bipartite zinc-binding module connecting these two domains, in a largely identical three-dimensional arrangement (22). We thus decided to re-examine the activity of human PRORP alone in comparison to that of the mtRNase P holoenzyme more thoroughly and on a wider set of human mitochondrial pre-tRNA substrates (Supplementary Figures S1A and S2). Confirming our previous observation (9), pre-tRNA^Ile^ and pre-tRNA^Tyr^, as well as several other mitochondrial pre-tRNAs were not specifically cleaved by PRORP alone even at an enzyme concentration 25-fold higher than the one originally used (Figure 1A and Supplementary Figure S6A). However, in the case of 5 out of the 10 newly tested mitochondrial pre-tRNAs, we detected substantial RNase P activity by PRORP alone, particularly at elevated concentrations (Figure 1B and Supplementary Figure S6B); notably, PRORP’s ability to independently cleave was not restricted to the two pre-tRNAs not methylated by TRMT10C–SDR5C1 (i.e. pre-tRNA^Met^ and pre-tRNA^Ser(UCN)^, which lack the otherwise ubiquitous target purine at position 9; see also Supplementary Figure S2), but includes also pre-tRNAs that are substrates for methylation by TRMT10C-SDR5C1 (i.e. pre-tRNA^Ala^, pre-tRNA^Glu^, pre-tRNA^His^). Nevertheless, in all those cases (including the two non-methylatable tRNAs) the addition of TRMT10C and SDR5C1 markedly increased the RNase P activity of PRORP. The latter observation is consistent with the strict requirement of all three proteins for efficient 5′-end processing of mitochondrial tRNAs *in vivo*, as previously demonstrated by the accumulation of mitochondrial pre-tRNAs upon knockdown of either TRMT10C, SDR5C1 or PRORP (9). Taken together, these findings indicate that human PRORP harbors a complete, functional active site and is able to recognize, bind, and cleave at least certain pre-tRNAs at the correct site on its own *in vitro*, whereas TRMT10C–SDR5C1 appear primarily required to increase the cleavage efficiency of PRORP.

**Figure 2.**
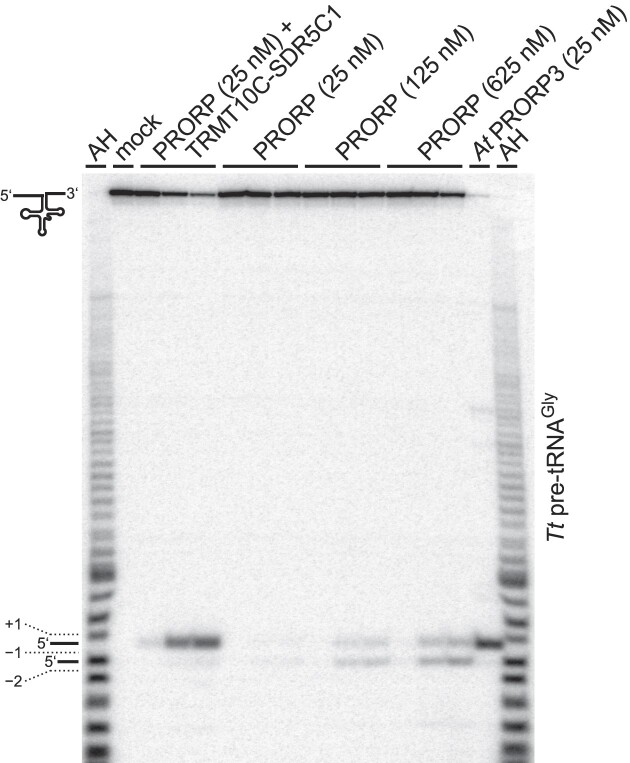
(Mis-)cleavage of an RNase P reference substrate by human PRORP. The RNase P activity of human PRORP was tested on bacterial *T. thermophilus* (*Tt*) pre-tRNA^Gly^. Aliquots were withdrawn from the reactions after 3, 30 and 60 min, and analyzed as described for Figure 1. A processing reaction with *A. thaliana* PRORP3 (*At* PRORP3) was included to identify the position of the canonical cleavage site between +1 and −1. In the first and last lanes, a nucleotide-resolution ladder, generated from pre-tRNA^Gly^ by partial alkaline hydrolysis (AH), was loaded to determine whether the observed extra cleavage occurred one or two nucleotides upstream of the canonical cleavage site. (Mis-)cleavage between pre-tRNA nucleotides −1 and −2 resulted in a one-nucleotide shorter 5′-leader product.

Animal mitochondrial tRNAs are structurally heterogeneous, deviate from canonical tRNA structures in the size of their D and TΨC loops, are characterized by a low number of G-C base pairs in their stems, and typically lack one or more of the common tertiary interaction motifs (ref. 44; see also Supplementary Figure S2). In addition to 12 mitochondrial pre-tRNAs, we also tested the processing of a fully canonical pre-tRNA by PRORP in the presence or absence of TRMT10C-SDR5C1. We chose pre-tRNA^Gly^ from *T. thermophilus*, a well-characterized bacterial model substrate that was previously used in a large number of studies with a wide variety of RNase P enzymes, including various PRORP homologs (15,30,45–52). The mtRNase P holoenzyme cleaved the bacterial pre-tRNA^Gly^ at the canonical cleavage site between position +1 and −1, like *A. thaliana* PRORP3 and any other previously tested form of RNase P (Figure 2). However, human PRORP alone not only showed weaker cleavage, but about 50% of the cleavage occurred one nucleotide upstream, between −1 and −2, in addition to the canonical site (Figure 2). These results suggest that TRMT10C–SDR5C1 may also affect the correct positioning of the target phosphodiester bond in the active site of PRORP, and that without the aid of TRMT10C-SDR5C1, PRORP’s ability to correctly select the cleavage site on some pre-tRNAs may be impaired.

### The effect of TRMT10C–SDR5C1 on pre-tRNA processing is specific to PRORP

The TRMT10C–SDR5C1 complex was previously suggested to act as a general tRNA-maturation platform in mitochondria, a concept based on the observation that besides being a mtRNase P subunit it also appeared to stimulate the cleavage by mitochondrial RNase Z (ELAC2) (53). While postulating a general role of TRMT10C–SDR5C1 in facilitating mitochondrial tRNA processing appears attractive, a common underlying mechanism would either require specific protein-protein interactions of largely the same TRMT10C surfaces with both, PRORP and ELAC2, or a more indirect, probably tRNA structure-mediated effect of TRMT10C-SDR5C1 to similarly stimulate the cleavage by the different effector nucleases. We re-assessed the stimulatory effect of TRMT10C–SDR5C1 on the RNase Z activity of ELAC2, and explored the possibility that small rearrangements of the tRNA structure by TRMT10C–SDR5C1 (or a related methyltransferase) per se may stimulate cleavage.

**Figure 3.**
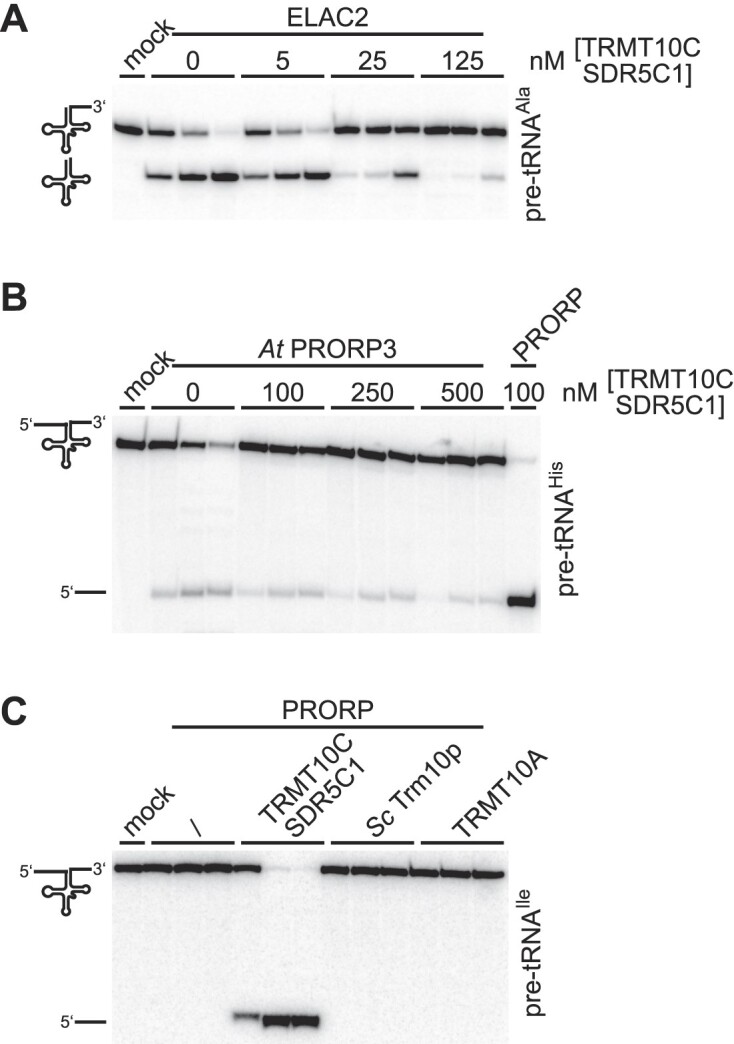
TRMT10C-SDR5C1 inhibits the activity of ELAC2 and *A. thaliana* PRORP3, and cannot be replaced by other TRM10 methyltransferases in mtRNase P reactions. (**A**) The effect of the TRMT10C–SDR5C1 complex on the RNase Z activity of ELAC2 was tested. Mitochondrial pre-tRNA^Ala^ with mature 5′ end was supplemented with the indicated concentrations of TRMT10C–SDR5C1 and tested for 3′ cleavage by His-ELAC2 (25 nM); aliquots were withdrawn at 1, 5 and 60 min, and analyzed as described for Figure 1. (**B**) The ability of the TRMT10C-SDR5C1 complex to stimulate RNase P cleavage was tested with a plant PRORP homolog. Human mitochondrial pre-tRNA^His^ was supplemented with the indicated concentrations of human TRMT10C–SDR5C1, and tested for cleavage by *A. thaliana* PRORP3 (*At* PRORP3; 100 nM); aliquots were withdrawn at 3, 30, and 60 min, and analyzed as described for Figure 1. (**C**) The RNase P activity of human PRORP (25 nM) was tested on human mitochondrial pre-tRNA^Ile^ in the presence of different methyltransferases of the TRM10 family: the human mitochondrial TRMT10C-SDR5C1 complex, *S. cerevisiae* Trm10p (*Sc* Trm10p), and human nuclear TRMT10A, each at a final concentration of 250 nM. Aliquots were withdrawn after 0.25, 15 and 60 min, and analyzed as described for Figure 1.

**Figure 4.**
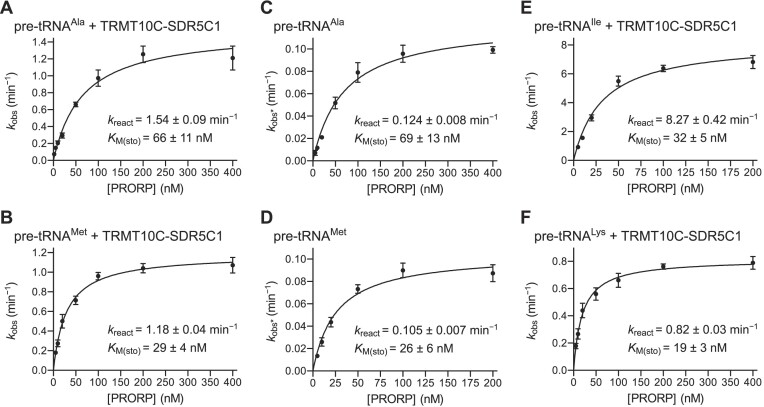
Single-turnover kinetics of pre-tRNA cleavage by the mtRNase P holoenzyme and human PRORP alone. Single-turnover kinetic analyses of pre-tRNA cleavage were performed with varying concentrations of PRORP in the presence of an excess of TRMT10C–SDR5C1 complex (mtRNase P holoenzyme; panels **A**, **B**, **E** and **F**) or PRORP alone (panels **C** and **D**). Pseudo first-order rate constants of cleavage (*k*_obs_ or *k*_obs*_; see Materials and Methods, and Supplementary Figure S3 and S4) were plotted against the concentration of PRORP. Data points are the mean ± SEM of at least five replicates. Derived kinetic constants *k*_react_ and *K*_M(sto)_ (best-fit values ± curve-fit standard error) are inserted into each graph. (**A**, **B**) Kinetic analysis of the cleavage of (**A**) pre-tRNA^Ala^ (at 3 mM Mg^2+^) and of (**B**) pre-tRNA^Met^ (at 4.5 mM Mg^2+^) by PRORP in the presence of TRMT10C–SDR5C1. (**C**, **D**) Kinetic analysis of the cleavage of (**C**) pre-tRNA^Ala^ (at 3 mM Mg^2+^) and of (**D**) pre-tRNA^Met^ (at 4.5 mM Mg^2+^) by PRORP alone. (**E**, **F**) Kinetic analysis of the cleavage of (**E**) pre-tRNA^Ile^ (at 3 mM Mg^2+^) and of (**F**) pre-tRNA^Lys^ (at 4.5 mM Mg^2+^) by PRORP in the presence of TRMT10C–SDR5C1.

In contrast to previous observations (53), TRMT10C–SDR5C1 inhibited, rather than stimulated, the RNase Z activity of ELAC2 in a dose-dependent manner (Figure 3A). This result was corroborated for 4 more mitochondrial pre-tRNAs, under multiple as well as single-turnover conditions, under higher ionic strength (as used in the previous study), and with recombinant ELAC2 tagged at either the C- or the N-terminus (Supplementary Figure S7A–C). Noteworthy, the efficiency of pre-tRNA^Ala^ cleavage by ELAC2 alone (Supplementary Figure S7D) resembled more that of the mtRNase P holoenzyme than that of PRORP alone (compare next section, Figure 4).

Likewise, TRMT10C–SDR5C1 did not stimulate, but slightly inhibited pre-tRNA 5′-end cleavage by *A. thaliana* PRORP3 (Figure 3B). Furthermore, attempts to replace the TRMT10C–SDR5C1 complex in its mtRNase P function by related methyltransferases, supposed to similarly remold the bound pre-tRNA, failed. When we replaced TRMT10C–SDR5C1 in our RNase P assays with *S. cerevisiae* Trm10p or human TRMT10A, both evolutionarily and structurally related to TRMT10C and able to methylate mitochondrial pre-tRNA^Ile^*in vitro* (19), neither of them rendered pre-tRNA^Ile^ cleavable by human PRORP (Figure 3C). Collectively, all these results suggest a high specificity in the interplay of PRORP with the TRMT10C-SDR5C1 complex, a specificity that appears mainly determined by the direct molecular interactions of PRORP and TRMT10C.

### TRMT10C-SDR5C1 increase the cleavage rate of PRORP

The finding that PRORP alone is able to correctly cleave certain pre-tRNAs allowed us to quantitatively assess the contribution of TRMT10C–SDR5C1 to the cleavage process by comparative kinetic analyses. We studied the kinetics of cleavage by the mtRNase P holoenzyme and by PRORP alone under single-turnover conditions (enzyme concentration in excess over substrate). Under these conditions, single rounds of catalysis occur, and product release and substrate-rebinding events do not contribute to the observed reaction rates, thereby putting the focus on the steps after the formation of the initial enzyme-substrate-encounter complexes (which includes the chemical step and possible conformational steps preceding it). We studied the cleavage kinetics of two mitochondrial pre-tRNA model substrates. In the case of the mtRNase P holoenzyme (PRORP + TRMT10C–SDR5C1), we measured a *K*_M(sto)_ of ∼70 nM and a maximal rate constant (*k*_react_) of ∼1.5 min^−1^ for pre-tRNA^Ala^ (Figure 4A), and a *K*_M(sto)_ of ∼30 nM and a *k*_react_ of ∼1.2 min^−1^ for pre-tRNA^Met^ (Figure 4B). With PRORP alone, we observed an ∼11- to 12-fold lower *k*_react_, but essentially identical *K*_M(sto)_ values for both pre-tRNAs (compare Figures 4B and D with 4A and B, respectively), suggesting that the TRMT10C-SDR5C1 complex increases the rate of conformational rearrangements and/or the chemical step, but not the affinity of PRORP for the substrates. In addition, we examined the mtRNase P holoenzyme-cleavage kinetics of three additional pre-tRNA substrates, pre-tRNA^Ile^, pre-tRNA^Lys^ and pre-tRNA^Val^, for which we observed no cleavage by PRORP alone (Figure 1). Interestingly, *K*_M(sto)_ and *k*_react_ were in the same order of magnitude as the corresponding values for pre-tRNA^Ala^ and pre-tRNA^Met^, or showed an even higher *k*_react_ in the case of pre-tRNA^Ile^ (Figure 4E and F; Supplementary Figure S8), indicating that the pre-tRNAs that are cleaved by PRORP alone are not necessarily better substrates for the mtRNase P holoenzyme. In this context, we also like to note the large variation of *k*_react_ values in holoenzyme reactions, exemplified by ∼0.8 min^−1^ for pre-tRNA^Lys^ or pre-tRNA^Val^ on the slow, and 8.3 min^−1^ for pre-tRNA^Ile^ on the fast end of the measured range; furthermore, preliminary kinetic experiments with pre-tRNA^Tyr^ or pre-tRNA^His^ indicated even higher rates of cleavage of >10 min^−1^, in fact too fast for reliable kinetics by manual sampling under the employed conditions.

### PRORP binds pre-tRNAs with high affinity

The low nanomolar *K*_M(sto)_ for pre-tRNA cleavage by PRORP, essentially independent of and unchanged by TRMT10C–SDR5C1, suggests that PRORP itself binds pre-tRNAs with high affinity and that the TRMT10C-SDR5C1 complex does not significantly contribute to catalytic efficiency by enhancing PRORP’s substrate affinity. We used bio-layer interferometry to directly study pre-tRNA binding by the subunits of mtRNase P. We immobilized the pre-tRNA on the sensor, and followed the association of the different proteins and their dissociation upon dilution. Specifically, we assayed the binding of TRMT10C, SDR5C1, and PRORP, to pre-tRNA^Ala^, pre-tRNA^His^, pre-tRNA^Lys^ and pre-tRNA^Val^ (representative curves for the binding to pre-tRNA^Lys^ are shown in Supplementary Figure S9), and derived the association (*k*_on_) and dissociation (*k*_off_) rate constants, as well as the resulting dissociation constant (*K*_D_) for each combination (Table 1). TRMT10C and PRORP showed pre-tRNA binding with *K*_D_ values in the low nanomolar range, whereas we observed no binding of SDR5C1 to any of the tested pre-tRNAs, confirming that SDR5C1 alone is not able to bind pre-tRNAs directly, as previously observed (19) and consistent with the structural information (22). The addition of SDR5C1 to TRMT10C nevertheless slightly lowered the observed *K*_D_ for all tested pre-tRNAs, mostly due to a reduction of *k*_off_, suggesting that the interaction with SDR5C1 somewhat stabilizes the binding of TRMT10C to the pre-tRNA substrate; still, this stabilizing effect is insufficient to explain the previously observed dramatic stimulation of TRMT10C’s methylation activity upon supplementation with SDR5C1 (19). Finally, the *K*_D_ of PRORP for pre-tRNAs was in a similar range of that of TRMT10C and of PRORP’s *K*_M(sto)_ for the cleavage reactions, and did not correlate with the ability of PRORP to cleave or not cleave a given pre-tRNA substrate on its own. The similarly high affinity of TRMT10C–SDR5C1 and PRORP for pre-tRNAs unfortunately made analogous binding studies with the mtRNase P holoenzyme pointless; moreover, preliminary bio-layer interferometry measurements with mixtures of all three proteins gave no meaningful results.

**Table 1.** Pre-tRNA binding parameters of the components of mtRNase P

	PRORP			TRMT10C			TRMT10C + SDR5C1		
pre-tRNA	*k*_on_ (M^−1^ min^−1^)	*k*_off_ (min^−1^)	*K*_D_ (nM)	*k*_on_ (M^−1^ min^−1^)	*k*_off_ (min^−1^)	*K*_D_ (nM)	*k*_on_ (M^−1^ min^−1^)	*k*_off_ (min^−1^)	*K*_D_ (nM)
Ala	5.7e^5^ ± 2.6e^4^	8.5e^−3^ ± 5.2e^−4^	15	1.2e^6^ ± 1.4e^4^	3.8e^−2^ ± 6.6e^−4^	32	2.2e^6^ ± 9.6^3^	3.2e^−2^ ± 3.4^−4^	15
His	1.1e^6^ ± 2.6e^4^	2.5e^−2^ ± 7.2e^−4^	23	3.3e^6^ ± 2.8^4^	4.1e^−2^ ± 6.0e^−4^	12	3.4e^6^ ± 1.6e^4^	2.9e^−2^ ± 3.4e^−4^	8
Lys^a^	7.2e^5^ ± 2.1e^4^	1.9e^−2^ ± 4.4e^−4^	26	2.0e^6^ ± 1.3e^4^	5.9e^−2^ ± 5.1e^−4^	30	1.5e^6^ ± 9.6e^3^	6.6e^−3^ ± 2.5e^−4^	4
Val	5.9e^5^ ± 8.7e^3^	1.0e^−2^ ± 9.9e^−4^	18	2.6e^6^ ± 2.5e^4^	5.0e^−2^ ± 1.3e^−3^	20	2.6e^6^ ± 1.2e^4^	3.4e^−2^ ± 4.8e^−4^	13

The pre-tRNA-binding parameters of the subunits of mtRNase P were determined by bio-layer interferometry. Parameters are reported as best-fit values ± curve-fit standard error. The dissociation constant (*K*_D_) was calculated as the ratio *k*_off_/*k*_on_. No binding signal above background was detected in the case of SDR5C1 alone even at 1 μM with any of the pre-tRNAs tested.

^a^Pre-tRNA^Lys^-binding was tested in buffer containing 4.5 mM Mg^2+^.

### PRORP does not require TRMT10C-SDR5C1 or pre-tRNA to properly coordinate Mg^2+^ in its active site

**Figure 5.**
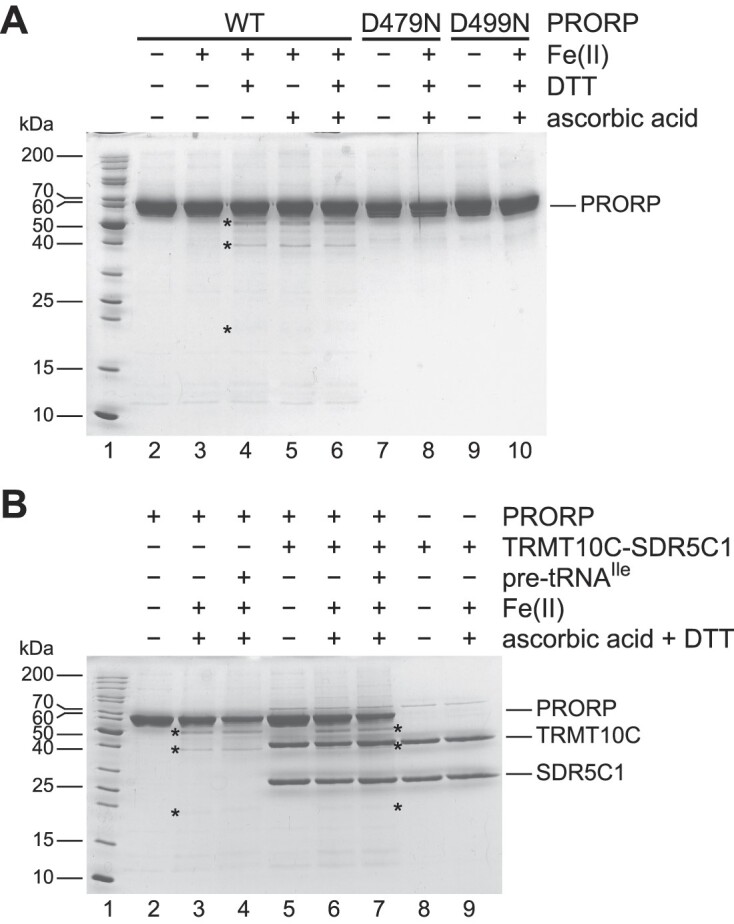
Metal-ion coordination in the active site of human PRORP. (**A**) PRORP was subjected to iron-mediated hydroxyl radical cleavage. The wild-type protein, or substitution variants D479N and D499N, were incubated with Fe(II), DTT and/or ascorbic acid, and cleavage products were resolved by SDS-PAGE; the gel was stained with Coomassie blue. Specific cleavage products in lanes 4–6 are indicated by asterisks (typically, only a small fraction of the protein is cleaved by this procedure, as previously demonstrated for other, well-established metalloenzymes; refs. 43,63); the barely visible ∼20 kDa fragment and the second, smaller complementary C-terminal fragment (not detectable by direct staining) were confirmed by western blotting (Supplementary Figure S10). The molecular weight of selected size markers (lane 1) is indicated on the left. The position of full-length PRORP is indicated on the right. (**B**) PRORP was subjected to iron-mediated hydroxyl radical cleavage in the presence of pre-tRNA^Ile^ (lane 4) and/or the TRMT10C-SDR5C1 complex (lanes 7 and 6). Specific cleavage products in lanes 3, 4 and 6, 7, are indicated by asterisks (note that the ∼40-kDa PRORP fragment migrates very close to TRMT10C in lanes 6 and 7, and can only be seen when zooming into the relevant area). The positions of full-length PRORP, TRMT10C and SDR5C1 are indicated on the right.

Our comparative kinetic analysis and pre-tRNA-binding studies suggested that, while PRORP is able to efficiently bind pre-tRNAs on its own, its interaction with TRMT10C-SDR5C1 is primarily required for some kind of ‘activation’ through structural changes in—and/or by repositioning of—its nuclease domain leading to significant rate enhancements and making some pre-tRNAs cleavable in the first place. Such a scenario appears at first sight consistent with a model that had been proposed on the basis of the crystal structures of PRORP fragments (28,29). In these structures, the active site of PRORP appears distorted and incapable of coordinating Mg^2+^, and the authors proposed that the interaction with TRMT10C–SDR5C1 would remodel the active site to restore Mg^2+^ coordination by PRORP and hence its endonuclease activity. Our finding that PRORP alone is able to cleave some pre-tRNAs, even though with reduced efficiency, appears difficult to reconcile with a strict requirement for TRMT10C–SDR5C1 to enable coordination of the catalytic metal ions. Therefore, we used iron-mediated hydroxyl radical cleavage (43) to directly probe whether PRORP alone is able to coordinate divalent metal ions in its active site. When PRORP was incubated with Fe(II) and a reducing agent, the protein underwent partial fragmentation producing polypeptides of about 50, 40, 20 and 12 kDa (Figure 5A, lanes 4, 5, 6; the smaller C-terminal fragments could only be visualized by western blotting using an antibody against the C-terminal His tag; see Supplementary Figure S10). This pattern is consistent with cleavage close to D409 and to one of the other three aspartate residues (D478, D479, D499) involved in coordination of the two catalytic metal ions (23,28,29). To confirm the specificity of the iron-mediated cleavage, we used substitution variants D479N and D499N; both aspartates are directly involved in metal-ion coordination in the cryo-EM structure of human mtRNase P and in the crystal structure of *A. thaliana* PRORP1 (22,23), but were reported to be rearranged and thereby unavailable for metal-ion coordination in the crystal structure of a human PRORP fragment (28). Both substitutions prevented iron-mediated cleavage (Figure 5A and Supplementary Figure S10, lanes 8 and 10), consistent with the involvement of the two aspartates in metal-ion coordination in human PRORP. An identical pattern and extent of iron-mediated fragmentation was observed when equimolar amounts of pre-tRNA^Ile^ or the TRMT10C–SDR5C1 complex, or both, were added to the reaction (Figure 5B, compare lanes 3, 4, 6 and 7). These results demonstrate that PRORP is able to coordinate metal ions in its active site irrespectively of an interaction with TRMT10C–SDR5C1 or pre-tRNA, consistent with its ability to also cleave at least some pre-tRNAs independently of TRMT10C–SDR5C1.

**Figure 6.**
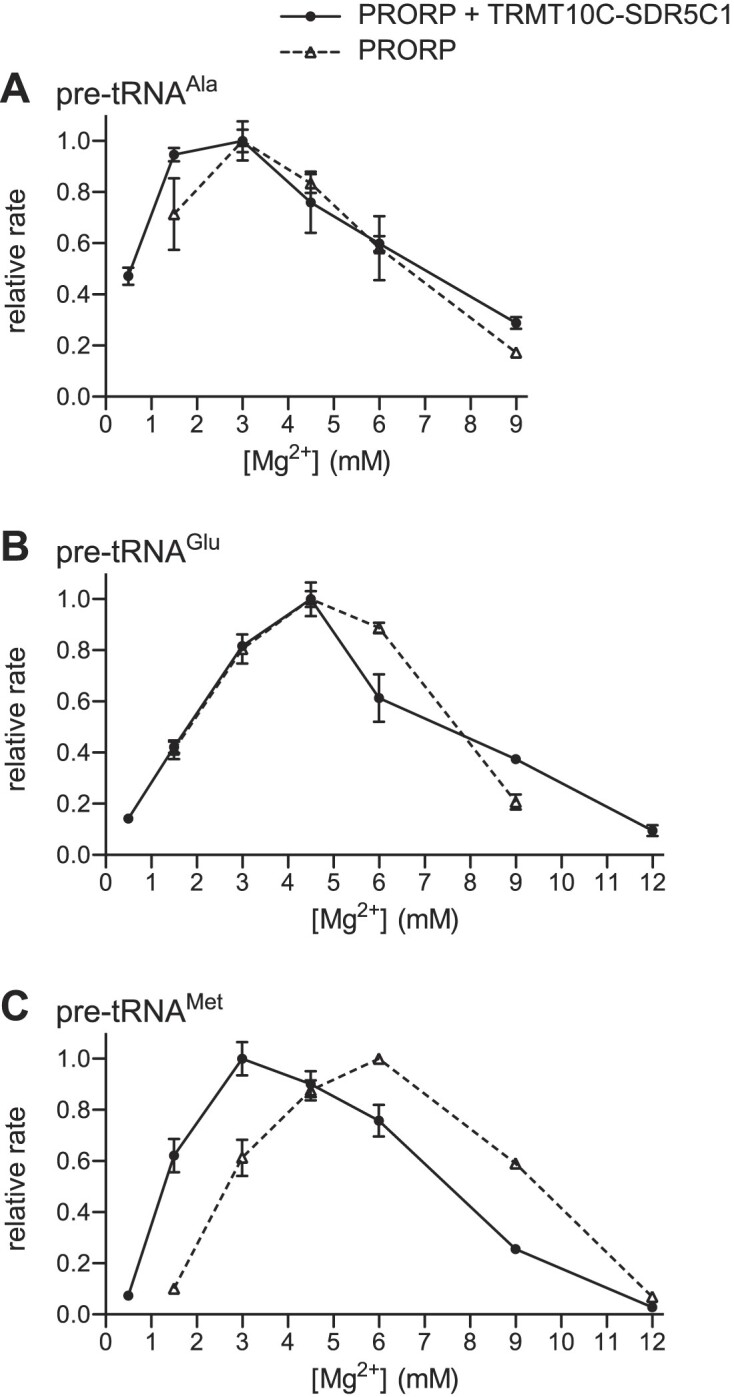
Mg^2+^ optimum of human PRORP compared to that of the mtRNase P holoenzyme. Cleavage kinetics were performed with PRORP alone and in the presence of TRMT10C-SDR5C1 (mtRNase P holoenzyme), at different Mg^2+^ concentrations under single-turnover conditions. Pseudo first-order cleavage rates (*k*_obs_ or *k*_obs*_) were derived and normalized to the highest rate measured. Relative rates represent the mean ± SEM of at least three replicates. Cleavage by PRORP alone at the lowest and/or highest Mg^2+^concentration shown for the mtRNase P holoenzyme was in several cases too weak to derive a corresponding cleavage rate, explaining the missing data points. (**A–C**) Mg^2+^ optima for the cleavage of (**A**) pre-tRNA^Ala^, (**B**) pre-tRNA^Glu^ and (**C**) pre-tRNA^Met^.

Higher Mg^2+^ concentrations could be expected to at least partially rescue an impaired coordination of Mg^2+^ in the active site, hence the Mg^2+^ optimum for cleavage by PRORP alone could be higher than that of the holoenzyme if such impairment were the case. Therefore, we also compared the Mg^2+^ optimum for cleavage by PRORP alone with that of the mtRNase P holoenzyme for three pre-tRNA model substrates. For pre-tRNA^Ala^ and pre-tRNA^Glu^ the highest cleavage rate was reached at the same Mg^2+^ concentration with both, PRORP and mtRNase P, albeit the optima for the two pre-tRNAs differed slightly (3 versus 4.5 mM; Figure 6A and B). In the case of pre-tRNA^Met^, the Mg^2+^ optimum of PRORP alone was twofold higher than that of mtRNase P (6 vs. 3 mM; Figure 6C). Overall, these results suggest that the affinity of human PRORP for divalent metal ions is not generally and systematically altered by the TRMT10C-SDR5C1 complex, although the Mg^2+^ optimum of PRORP and the holoenzyme may slightly differ in some cases.

### Product release is not a rate-limiting step in mtRNase P catalysis

**Figure 7.**
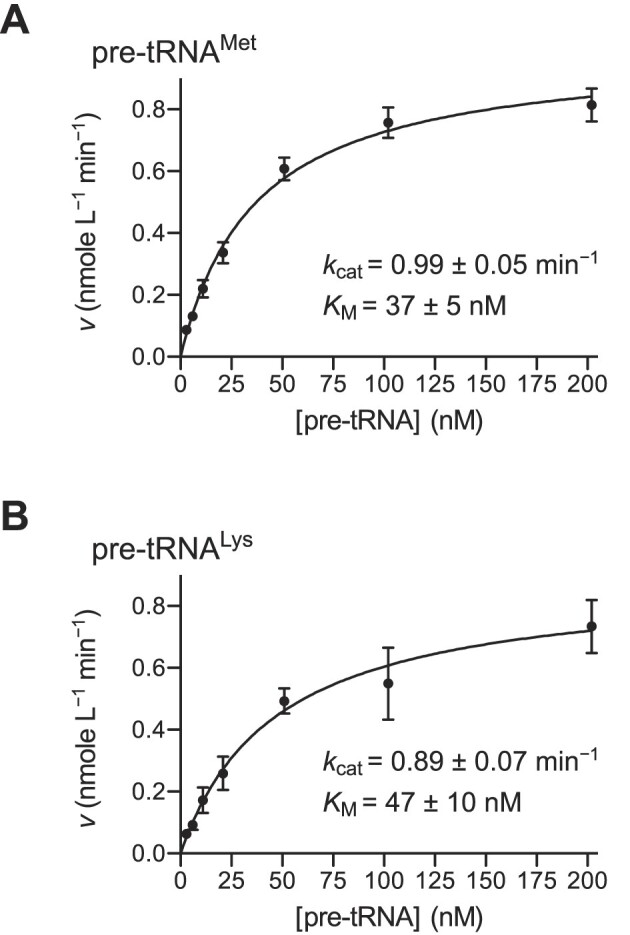
Multiple-turnover kinetics of pre-tRNA cleavage by mtRNase P. Multiple-turnover kinetic analyses of pre-tRNA cleavage were performed with 1 nM PRORP in the presence of an excess of TRMT10C-SDR5C1 complex and with varying concentrations of pre-tRNA (at 4.5 mM Mg^2+^). Initial velocities of cleavage (*v*; see Materials and Methods, and Supplementary Figure S5) were plotted against the pre-tRNA concentration. Data points are the mean ± SEM of at least five replicates. Derived kinetic constants *k*_cat_ and *K*_M_ (best-fit values ± curve-fit standard error) are inserted into each graph. (**A**, **B**) Multiple-turnover kinetic analysis of the cleavage of (**A**) pre-tRNA^Met^ and (**B**) pre-tRNA^Lys^ by mtRNase P.

To complete our studies of mtRNase P catalysis, we also performed experiments addressing the early and late steps of catalysis, i.e. steps preceding or following the chemical (cleavage) step, respectively. First, we studied the multiple-turnover kinetics of cleavage by mtRNase P to see, by comparison with single-turnover kinetics, whether product release may be rate-limiting. We again used saturating concentrations of TRMT10C-SDR5C1 and determined *K*_M_ and *k*_cat_ for two pre-tRNA substrates also analyzed under single-turnover conditions (see above and Figure 4). For both, pre-tRNA^Met^ and pre-tRNA^Lys^, we measured a *k*_cat_ almost identical to the *k*_react_ obtained under single-turnover conditions and a *K*_M_ only slightly higher than the *K*_M(sto)_ (Figure 7; compare to Figure 4B and F). These results essentially rule out that product release is the rate-limiting step under multiple-turnover conditions and suggest that either the chemical step or (a) preceding step(s) limit(s) the rate of cleavage.

**Figure 8.**
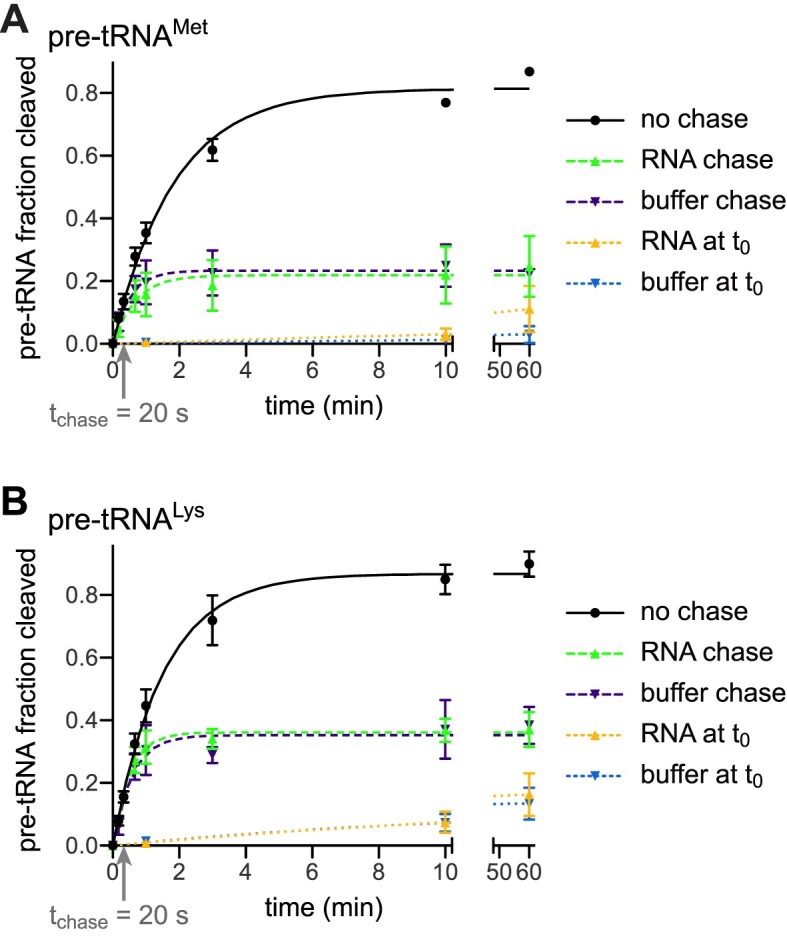
Pulse-chase kinetics of pre-tRNA cleavage by mtRNase P. Trace amounts of pre-tRNA were cleaved by an excess of mtRNase P (PRORP + TRMT10C-SDR5C1) at 4.5 mM Mg^2+^, and the reactions were quenched (‘chase’) after 20 s, either by addition of a 1000-fold excess of unlabeled substrate, or by 200-fold dilution with reaction buffer. Aliquots were withdrawn and the reaction stopped at the indicated time points. Samples were analyzed by gel electrophoresis and phosphorimaging, and the cleaved fraction of pre-tRNA was determined by image analysis. Control reactions without a ‘chase’ and with the ‘chase’ prior to the start of the reaction (*t*_0_) were run in parallel. Pulse-chase kinetic analysis of (**A**) pre-tRNA^Met^ and of (**B**) pre-tRNA^Lys^ cleavage by mtRNase P.

Finally, we performed pulse-chase experiments as introduced by the Uhlenbeck lab in their studies of hammerhead ribozymes (41), to shed light on the relationship between the dissociation rate constant (*k*_−1_) of pre-tRNA and mtRNase P, and the rate constant of the chemical step (*k*_2_) (according to this minimal kinetic model of cleavage by RNase P: ${\mathrm{E}} + {\mathrm{S}}\ \begin{array}{@{}*{1}{c}@{}} {{k}_1}\\ \rightleftharpoons \\ {{k}_{ - 1}} \end{array}{\mathrm{\ ES}}\ \begin{array}{@{}*{1}{c}@{}} {{k}_2}\\ \to \\ {} \end{array}\ {\mathrm{EP}}\ \begin{array}{@{}*{1}{c}@{}} {{k}_3}\\ \rightleftharpoons \\ {{k}_{ - 3}} \end{array}\ {\mathrm{E}} + {\mathrm{P}}$; conformational steps possibly preceding *k*_2_ are not taken into account here). Under single-turnover conditions with an excess of enzyme, cleavage reactions were quenched after a short period of reaction progress, either by addition of an excess of unlabeled substrate or by dilution with reaction buffer. Assuming all substrate is enzyme-bound at the time point of the chase, an unaltered progress of the reaction would indicate that *k*_2_ exceeds *k*_−1_ by far, whereas an immediate halt would indicate the reverse, i.e. *k*_−1_ ≫ *k*_2_. MtRNase P showed an intermediate behavior, with some cleavage continuing to occur after the chase (Figure 8). This intermediate kinetic behavior indicates that the rate of substrate dissociation and of chemistry are in a similar range, with *k*_2_ probably exceeding *k*_−1_, based on the dissociation rate constants measured for PRORP alone by bio-layer interferometry (Table 1; substrate dissociation still has to be assumed to be faster at the cleavage-assay temperature of 30°C relative to the 21°C of the bio-layer interferometry measurements).

## DISCUSSION

Since the initial identification of the protein-only RNase P found in human mitochondria (9), the diversity of its subunits and their additional role in other pathways have obscured the holoenzyme's mechanism of action and puzzled the field (10). The characterization of homologs of the TRM10 and PRORP families, all active as single proteins, only further underlined the exceptional status of those animal mtRNase P subunits within the respective enzyme families. While the recent cryo-EM structure of human mtRNase P elucidated the interactions of the individual subunits with each other and the pre-tRNA substrate (22), it did not shed light on the mechanistic interplay among the subunits, the dynamic aspects of the assembly of the enzyme-substrate complex, and the kinetics of the reaction. Moreover, the association of pathogenic mutations in the genes encoding the three subunits with largely distinct clinical presentations warrants closer attention to these aspects (54–56).

Here, we sought to more precisely define the mechanism of action of the catalytic subunit PRORP, and to shed light on its interplay with the ‘accessory’ subunits TRMT10C and SDR5C1 in achieving efficient phosphodiester hydrolysis. Unexpectedly, we found that PRORP alone is able to efficiently bind pre-tRNAs and to even cleave some of them, although at an at least 10-fold lower rate than in the presence of TRMT10C–SDR5C1. These findings suggest a model in which in principle the TRMT10C-SDR5C1 complex and PRORP are able to bind pre-tRNAs and exert catalysis independently of each other: TRMT10C–SDR5C1 by methylating the *N*^1^ of purines found at position 9 in 19 of the 22 mitochondrial tRNAs (19), and PRORP by removing the 5′ extension of at least some mitochondrial pre-tRNAs. Still, only the successive or coincident binding of PRORP and TRMT10C-SDR5C1 to a pre-tRNA, and the resulting direct molecular interactions between TRMT10C and PRORP lead to the efficient removal of the 5′ extension from pre-tRNAs, consistent with the strict requirement of all 3 subunits for efficient mtRNase P function *in vivo* (9). PRORP and TRMT10C-SDR5C1 bind to all tested pre-tRNAs with similar affinity and there is no evidence from the kinetic analyses that either the PRORP·pre-tRNA or the TRMT10C–SDR5C1·pre-tRNA complex represents the preferred pathway to the cleavage-efficient holoenzyme·pre-tRNA complex; the order of events *in vivo* may thus simply depend on the availability and relative stoichiometries of the two players PRORP and TRMT10C-SDR5C1.

In the light of evolution, the vestigial nuclease activity of human PRORP appears not too surprising, given its apparent descent from single-subunit PRORPs (10,18). Remarkably, human PRORP still appears to generally bind pre-tRNAs with similar affinity, regardless of whether it is able to cleave them on its own or not. TRMT10C-SDR5C1 exerts its effect on the cleavage kinetics of PRORP by a rate enhancement rather than by decreasing the *K*_M(sto)_, as inferred from the cases where PRORP alone is also able to cleave the substrate. This finding suggests that direct interactions with TRMT10C induce rearrangements in, and/or a re-positioning of, PRORP’s nuclease domain that allow the (more efficient) hydrolysis of the target phosphodiester bond. The interactions of PRORP’s PPR domain with the tRNA ‘elbow’ are probably not substantially affected by the interaction with TRMT10C and, considering the latter's kinetic effect, they likely remain similar or even identical in the holoenzyme.

Conformational changes as a mechanism in the activation of human PRORP were previously proposed based on the crystal structures of PRORP fragments (28,29). The interaction with TRMT10C-SDR5C1 was suggested to remold the observed disordered catalytic domain of PRORP to enable the coordination of Mg^2+^ in its active site. However, our experiments show that PRORP can actually coordinate divalent metal ions irrespectively of the presence of TRMT10C-SDR5C1 or pre-tRNA, and the ability of PRORP to cleave a subset of pre-tRNAs on its own also demonstrates that it properly coordinates the essential cofactor Mg^2+^ in its active site; in addition, neither we nor others (57) observed a systematic enhancement of the Mg^2+^ affinity of PRORP by TRMT10C-SDR5C1. This suggests that the rearrangements observed in the crystals of PRORP are either an artifact (28,29) and do not represent a physiologically relevant conformation of the nuclease, or they are a consequence of the absence of metal ions in the crystals. The latter appears consistent with the absence of metal ion 1 in the cryo-EM structure and a D478 side chain being tilted away (‘rearranged’) from its metal-coordinating position (22) when compared to the corresponding D474 side chain in the *A. thaliana* PRORP1 crystal structure, which contains both metal ions (23). Still, the disparate steric arrangement of D478 and D479 in the two PRORP crystals also casts doubts on the significance of the structures, and particularly the rearrangements of the central domain could be the artifactual result of the truncated PPR domain of the crystallized fragments (28,29); notably, those fragments were catalytically inactive even in the presence of TRMT10C–SDR5C1. In the end, our results demonstrate that the nuclease domain of PRORP is in principle functional (on its own) and a restoration of the ability to coordinate Mg^2+^ is apparently not the principle underlying the strong ‘activating’ effect of TRMT10C–SDR5C1.

The favorable positioning or locking-into-position of PRORP’s nuclease domain on the tRNA’s acceptor stem to achieve its proper adjustment to the cleavage site rather appears to be the major conformational adjustment(s) that is induced by the interaction with TRMT10C; simultaneously, TRMT10C interacts with PRORP’s PPR domain and holds it in place (22). Although the ancient binding mode of single-subunit PRORPs has apparently been at least partially retained by the human homolog, as indicated by the reasonably high affinity with which it binds pre-tRNAs, it no longer firmly coaxes into efficient and specific catalysis. Depending on the respective substrate, this manifests as no, low, or aberrant cleavage activity by PRORP alone. Substantial miscleavage of *T. thermophilus* pre-tRNA^Gly^ by PRORP alone, but not by the mtRNase P holoenzyme, supports such a model. The subtle rearrangement of the TRMT10C-SDR5C1-bound tRNA structure observed in the cryo-EM structure (22), might be irrelevant for the cleavage process in this scenario, and the direct interactions of TRMT10C with PRORP’s nuclease domain appear to be the major driver of the conformational adjustments that boost specific and efficient cleavage by PRORP.

Even with its most efficiently cleaved substrates, human PRORP on its own is a poor catalyst and ≳10-fold slower than in the presence of an excess of TRMT10C-SDR5C1. With rates of ∼1 min^−1^ on most of the tested substrates, the mtRNase P holoenzyme is still comparably slow, yet some mitochondrial pre-tRNAs (e.g. pre-tRNA^Ile^, and apparently also pre-tRNA^His^ or pre-tRNA^Tyr^) are cleaved at ∼10-fold higher rates by the holoenzyme, comparable to the rates observed with *A. thaliana* PRORP3 on canonical pre-tRNA substrates (30). In fact, this remarkably broad range of maximal rate constants (*k*_react_) observed in single-turnover kinetics suggests a substrate-dependent rate-limiting step following enzyme-substrate complex formation.

Comparison of the kinetics of cleavage by the mtRNase P holoenzyme revealed that single-turnover *k*_react_ values and the turnover number (*k*_cat_) derived from multiple-turnover analyses were almost identical for the two substrates analyzed. Thus, similar to *A. thaliana* PRORP1 (58), product release is not a rate-limiting step in the catalysis by human mtRNase P. Pulse-chase experiments and the broad range of *k*_react_ values combined with similar *K*_M(sto)_ and *K*_D_ values, rather suggest that some (conformational) step between initial binding and cleavage limits reaction velocity in a substrate-dependent manner. By itself, the observation that the chemical step (*k*_2_) and the dissociation rate (*k*_−1_) are apparently in a similar range, does not necessarily indicate a conformational step preceding phosphodiester hydrolysis. However, the observed wide range of *k*_react_ values can hardly be explained by differences in *k*_2_ alone. Differences at the cleavage site, like base identity immediately upstream of the hydrolyzed phosphodiester bond, affect the chemical step, as for *A. thaliana* PRORP3 (30), by less than a factor of three (Hazisllari and Rossmanith, unpublished observations). Yet, no features in close proximity of the cleavage site are recognizable in the studied pre-tRNAs that may explain the broad range of cleavage rates. Taken together, the findings rather suggest that an additional, conformational step (or several thereof) before the chemical step is affected and varies between the different substrates, which appears consistent with the suggested conformational adjustments of the nuclease domain on the substrate through its interaction with TRMT10C. The features of a pre-tRNA that make it pass through those conformational steps slower or faster remain unclear, though, and will have to await future studies directly addressing this issue (see also below).

In an independent mechanistic study of human mtRNase P based on kinetic and binding experiments and available as a preprint, the binding affinity of the mtRNase P holoenzyme for pre-tRNA, as measured by fluorescence anisotropy, was reported to be greater than that of the separate subunits (57). Based on that finding and the kinetic enhancement of cleavage by increasing concentrations of TRMT10C–SDR5C1, the authors proposed that TRMT10C–SDR5C1 contributes to cleavage by increasing the binding affinity of PRORP in addition to a rate enhancement. Although we could not directly quantify pre-tRNA binding of the holoenzyme, our kinetic data do not indicate such an effect, as the *K*_M(sto)_ of PRORP in the cleavage of two different pre-tRNA substrates was unaffected by TRMT10C–SDR5C1. However, the pre-tRNA affinity of PRORP that we measured by bio-layer interferometry was substantially (almost 100-fold) higher than in their case and they also did not observe cleavage by PRORP alone. While the latter difference might be attributable to the specific bacterial pre-tRNA substrate they used, the affinity of PRORP for pre-tRNAs cleaved by PRORP alone and for those requiring TRMT10C–SDR5C1 was similar in our hands.

The TRMT10C–SDR5C1 complex was previously suggested to also stimulate pre-tRNA cleavage by mitochondrial RNase Z (ELAC2) (53). We could not reproduce this observation, but rather found a consistent inhibition of the RNase Z activity of ELAC2 under all the conditions tested. In addition, ELAC2 per se behaved kinetically efficient and comparable to the mtRNase P holoenzyme rather than to PRORP alone, further questioning a physiological need for an accessory subunit like TRMT10C–SDR5C1. Finally, in contrast to the mtRNase P subunits, ELAC2 is dually localized and responsible for the endonucleolytic 3′-end processing of nucleus-encoded tRNAs too (5,6,59), evidently in the absence of TRMT10C–SDR5C1.

We did not directly address the methylation or cleavage kinetics of TRMT10C–SDR5C1 in this study, but preliminary attempts to detect a multiple-turnover ability failed, consistent with observations previously reported by others (53). Such a lack of multiple turnover capacity is surprising, given that the dissociation rate constants for TRMT10C–SDR5C1 were on average not significantly lower than those for PRORP under the same conditions. The mechanistic basis of the lacking multiple-turnover ability, and how the release of products (5′-processed and/or methylated pre-tRNAs) from TRMT10C-SDR5C1 is achieved, remain unclear. However, the inhibitory effect on RNase Z suggests that product release by TRMT10C–SDR5C1 would be advantageous or required for efficient 3′ processing to occur, as the processing of mitochondrial pre-tRNAs is ordered, with 5′ processing preceding that at the 3′ end *in vitro* and *in vivo* (5,7,59,60). We can yet not exclude that RNase Z may act with reduced efficiency on 5′-mature pre-tRNAs while they are still bound to TRMT10C–SDR5C1.

As discussed above, TRMT10C–SDR5C1 appears to enable or stimulate mtRNase P cleavage activity by adjusting PRORP’s nuclease domain and active site to the bound pre-tRNA via specific protein-protein interactions. The pre-tRNAs that are cleaved by PRORP alone might have a more favorable orientation of their domains due to stem and loop lengths, and/or may expose functional groups of key nucleotides such that they can basically mediate correct positioning in the active site without the support of TRMT10C–SDR5C1. Alternatively, these tRNA structures might benefit from a particular flexibility, which allows them to transiently assume conformations more favorable for cleavage; indeed, human mitochondrial pre-tRNAs have rather unstable structures due to an elevated number of A-U base pairs, non-canonical base pairs and mismatches in their stems, and the frequent lack of the conserved tertiary interactions (44), and, as a consequence, are predicted to assume multiple alternative conformations in solution (Vilardo, Wolfinger, Hofacker and Rossmanith, unpublished analysis). However, despite extensive comparisons of the pre-tRNAs that can be processed by PRORP alone *in vitro* with those that require TRMT10C–SDR5C1, we could neither establish a clear-cut correlation with some structural feature nor identify any signature indicative of one or the other. This might be due to the limited number of mitochondrial pre-tRNA substrates, yet the finding that *T. thermophilus* pre-tRNA^Gly^, a fully canonical tRNA with G-C rich stems and therefore a stable and probably more rigid structure than any mitochondrial tRNA, is a substrate for PRORP alone too, foreshadows that the reasons may not be simply explainable by a specific property of certain mitochondrial pre-tRNAs. In fact, the issue may lend itself to an undirected screening approach, like high-throughput sequencing kinetics (HTS-Kin; ref. 61), to parse the substrate recognition determinants of PRORP alone versus those of mtRNase P.

The mitochondrial methyltransferase TRMT10C–SDR5C1, which is only found in metazoans (62), has apparently been recruited as a general accessory factor to safeguard proper cleavage of the structurally degenerating pre-tRNA substrates by PRORP, like an extra recognition-handle beyond the direct contacts of PRORP with the pre-tRNA. This cooperation appears not only to have developed into a specific catalytic dependency of the nuclease on its interaction partner, but has apparently also maximally broadened the ‘substrate’ range of the methyltransferase complex to encompass even the pre-tRNAs it cannot methylate, as they do not have a purine at position 9.

## Supplementary Material

gkad713_Supplemental_file

## Data Availability

The data underlying this article are available in the article and in its online supplementary material. Primary data underlying enzyme kinetic analyses will be shared on reasonable request to the corresponding author.
