# Supplementary material for: Cleavage kinetics of human mitochondrial RNase P and contribution of its non-nuclease subunits
Source: Nucleic Acids Res. 2023 Oct 2;51(19):10536–50. doi: 10.1093/nar/gkad713 (PMC10602865; doi:10.1093/nar/gkad713)
Supplement: gkad713_Supplemental_file [file gkad713_supplemental_file.pdf]

SUPPLEMENTARY DATA

Cleavage kinetics of human mitochondrial RNase P and contribution of its non-nuclease subunits

Elisa Vilardo[1], Ursula Toth[1], Enxhi Hazisllari[1], Roland K. Hartmann[2] and Walter Rossmanith[1,*]

[1]Center for Anatomy & Cell Biology, Medical University of Vienna, 1090 Vienna, Austria

[2]Institute of Pharmaceutical Chemistry, Philipps-University Marburg, 35037 Marburg, Germany

[*]To whom correspondence should be addressed. Email: walter.rossmanith@meduniwien.ac.at

A

pre-tRNA^{Ala}

*GGGAGACCGGAAUU*CUAAGUGUUUGUGGGUUUAAGUCCCAUUGGUCUAGUAAGGGCUUAGCUUAAUUAAAGUGGCUGAUUUGCGUUCAGUUGAUGCAGAGUGGGGUUUUGCAGUCCUUAGCUGUUACAGAAAUUAAGUA*GGAUC*

pre-tRNA^{Cys}

GGUAAAAUGGCUGAGUGAAGCAUUGGACUGUAAAUCUAAAGACAGGGGUUAGGCCUCUUUUUACCAGCUCCGAGGUGAUUUUCAUAUUGAAUUGCAAAUUCGAAGAAGCAGCUUCAAACCUGCCGGGGCUUCUCCCGCCUUUUUUCCCGGCGGC*AUGCAAGCU*

pre-tRNA^{Gln}

*GGGAGACCGGAAUUC*AUGGGCCCGAUAGCUUAUUUAGCUGACCUUACUUUAGGAUGGGGUGUGAUAGGUGGCACGGAGAAUUUUGGAUUCUCAGGGAUGGGUUCGAUUCUCAUAGUCCUAGAAAUAAGGGGGUUUAAG*GAUC*

pre-tRNA^{Glu}

*GGGAGACCGGAAUU*CAUGCGGAGAUGUUGGAUGGGGUGGGGAGGUCGAUGAAUGAGUGGUUAAUUAAUUUUAUUAGGGGGUUAAUUUUGCGUAUUGGGGUCAUUGGUGUUCUUGUAGUUGAAAUACAACGAUGGUUUUUCAUAUCAUUGGUCGUGGUUGUAGUCCGUGCGAGAAUAAUGAUGUAUGCUUUGUUUCUGUUGAGUGUGGGUUUC*UAG*

pre-tRNA^{His}

*GGGAACAAAAGCUUGCAUGCCUGCAGGUCGACUCUAG*ACAUCAUUACCGGGUUUUCCUCUUGUAAAUAUAGUUUAACCAAAACAUCAGAUUGUGAAUCUGACAACAGAGGCUUACGACCCCUUAUUUACCGAGAAAGCUCACAAGAAU*U*

pre-tRNA^{Ile}

*GGGAGACCGGAAUU*CAAUCUCCAGCAUUCCCCCUCAAACCUAAGAAAUAUGUCUGAUAAAAGAGUUACUUUGAUAGAGUAAAUAAUAGGAGCUUAAACCCCCUUAUUUCUAGGACUAUGAGAAUCGAAC*CCUCUAGAGUCGA*

pre-tRNA^{Lys}

*GGGAGACCGGAAUUC*CGUAUUUACCCUAUAGCACCCCCUCUACCCCCUCUAGAGCCCACUGUAAAGCUAACUUAGCAUUAACCUUUUAAGUUAAAGAUUAAGAGAACCAACACCUCUUUACAGUGAAAUGCCCCAAC*GGAUC*

pre-tRNA^{Met}

*GGGAGACCGG*AAUUCUCCGUGCCACCUAUCACACCCCAUCCUAAAGUAAGGUCAGCUAAAUAAGCUAUCGGGCCCAUACCCCGAAAAUGUUGGUUAUACCCUUCCCGUACUAAUUAAUCCCCUGGCCCAACCCGUCAUCUACUCUA*GAGUCGA*

pre-tRNA^{Pro}

*GGGAGACCCAAG*CUUGGGUGGUACCCAAAUCUGCUUCCCCAUGAAAGAACAGAGAAUAGUUUAAAUUAGAAUCUUAGCUUUGGGUGCUAAUGGUGGAGUUAAAGACUUUUUCUCUGAUUUGUCCUUGGAAAAAGGUUUUCAUCUCCG

pre-tRNA$^{Ser(UCN)}$

*GGGAGACCGGAAUU*CCUUUUUGAAAAAGUCAUGGAGGCCAUGGGGUUGGCUUGAAACCAGCUUUGGGGGGUUCGAUUCCUUCCUUUUUUGUCUAG*GAUC*

pre-tRNA^{Tyr}

*GGGAGACCGGAAUUC*UAGAGAAUAGUCAACGGUCGGCGAACAUCAGUGGGGGUGAGGUAAAAUGGCUGAGUGAAGCAUUGGACUGUAAAUCUAAAGACAGGGGUUAGGCCUCUUUUUACCAGCUCCGAGGUGAUUUUCAUAUUGAAUUGCAAAUU*GGAUC*

pre-tRNA^{Val}

*GGGAGACCGGAAUUCGAGCUC*UAAGUGUACUGGAAAGUGCACUUGGACGAACCAGAGUGUAGCUUAACACAAAGCACCCAACUUACACUUAGGAGAUUUCAACUUAACUUGACCGCUCUGAGCUAAACCUAGCCCCAAACCCACUCCACCUUA*UCUAG*

B

pre-tRNA^{Ala}

AAGGGCUUAGCUUAAUUAAAGUGGCUGAUUUGCGUUCAGUUGAUGCAGAGUGGGGUUUUGCAGUCCUUAGCUGUUACAGAAAUUAAGUA*GGAUC*

pre-tRNA^{His}

GUAAAUAUAGUUUAACCAAAACAUCAGAUUGUGAAUCUGACAACAGAGGCUUACGACCCCUUAUUUACCGAGAAAGCUCACAAGAAU*U*

pre-tRNA^{Ile}

AGAAAUAUGUCUGAUAAAAGAGUUACUUUGAUAGAGUAAAUAAUAGGAGCUUAAACCCCCUUAUUUCUAGGACUAUGAGAAUCGAACCC

pre-tRNA$^{Leu(UUR)}$

GUUAAGAUGGCAGAGCCCGGUAAUCGCAUAAAACUUAAAACUUUACAGUCAGAGGUUCAAUUCCUCUUCUUAACAACAUACCCAUGGCCAACCUCCUACUCCUCAUUGU

pre-tRNA$^{Ser(UCN)}$

GAAAAAGUCAUGGAGGCCAUGGGGUUGGCUUGAAACCAGCUUUGGGGGGUUCGAUUCCUUCCUUUUUUGUCUAGAUUUUAUGUAUACGGU

Supplementary Figure S1. Sequences of the mitochondrial pre-tRNA substrates used in this study. (A) Complete sequence of the mitochondrial pre-tRNA transcripts used for RNase P activity assays; tRNA sequences in red, sequences derived from polylinker or cloning sites in italics. (**B**) Complete sequence of the pre-tRNA transcripts used for RNase Z activity assays; tRNA sequences in red, sequences derived from cloning sites in italics.

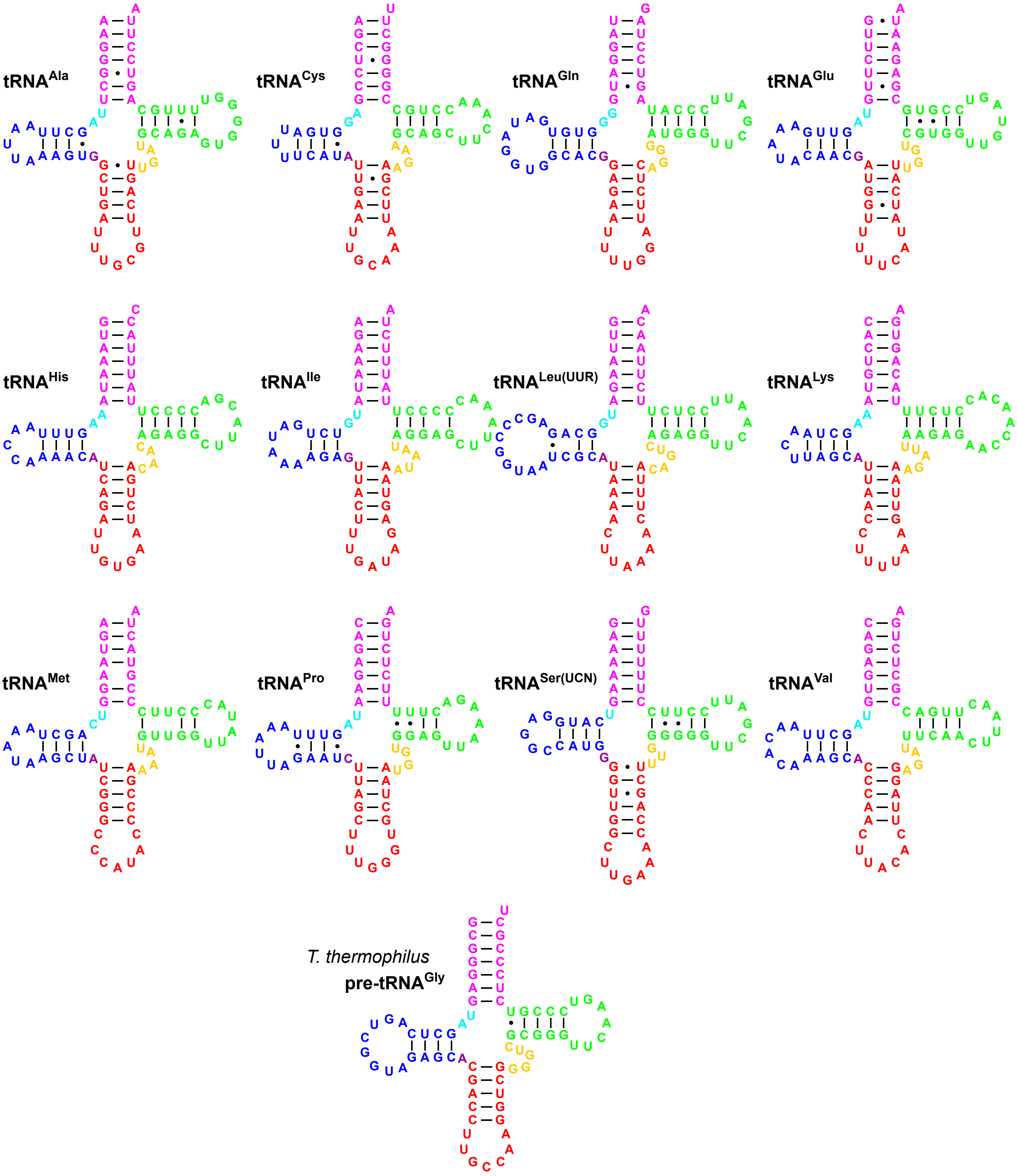

Supplementary Figure S2. Cloverleaf representation of the tRNA moieties of the substrates used in this study. The structural domains are color-coded: magenta, aminoacyl acceptor stem; cyan, connector; blue, D stem-loop; red, anticodon stem-loop; yellow, variable region; green, T stem-loop.

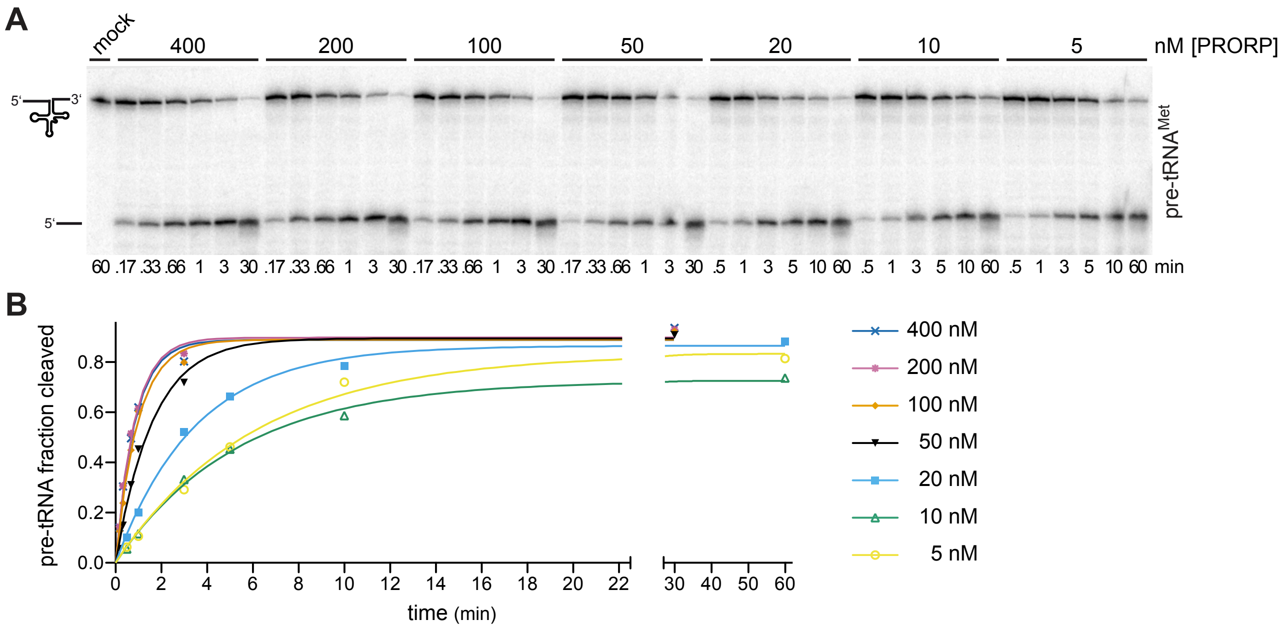

Supplementary Figure S3. Single-turnover kinetic analysis of cleavage by the mtRNase P holoenzyme (PRORP plus TRMT10C-SDR5C1); a representative example using mitochondrial pre-tRNA^{Met} as substrate. (**A**) Reactions with the indicated concentrations of PRORP were carried out as described in the Materials and Methods section of the main text (at 4.5 mM Mg^{2+}). Aliquots were withdrawn and the reaction stopped at the indicated time points. Samples were analyzed by gel electrophoresis and phosphorimaging. (**B**) Radioactive bands representing pre-tRNA and 5′-cleavage product were quantified to determine the cleaved substrate fraction. Data were fit by nonlinear regression analysis to determine pseudo first-order rate constant of cleavage (k_{obs}) at each PRORP concentration. These data were plotted in graphs as shown in Figure 4 and fit to a “Michaelis-Menten-like” enzyme kinetics model to derive k_{react} and $K_{M(sto)}$.

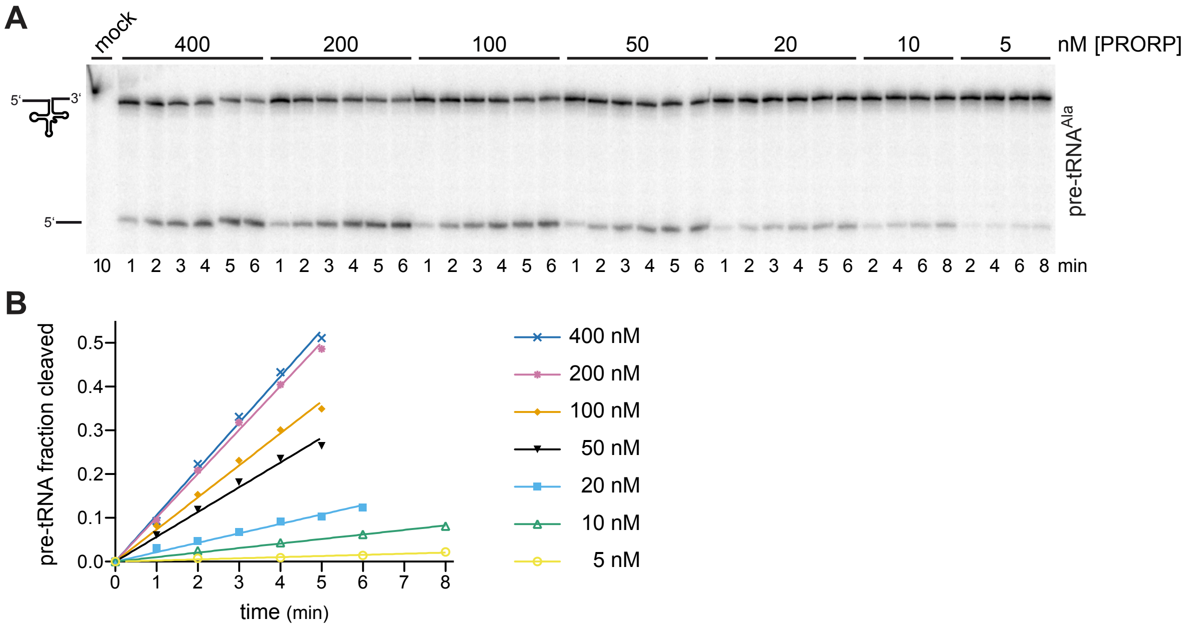

Supplementary Figure S4. Single-turnover kinetic analysis of cleavage by PRORP alone; a representative example using mitochondrial pre-tRNA^{Ala} as substrate. (**A**) Reactions with the indicated concentrations of PRORP were carried out as described in the Materials and Methods section of the main text (at 3 mM Mg^{2+}). Aliquots were withdrawn and the reaction stopped at the indicated time points. Samples were analyzed by gel electrophoresis and phosphorimaging. (**B**) Radioactive bands representing pre-tRNA and 5'-cleavage product were quantified to determine the cleaved substrate fraction. Data from the initial linear phase of the reaction were fit by linear regression analysis to approximate the pseudo first-order rate constant of cleavage (k_{obs*}) at each PRORP concentration. These data were plotted in graphs as shown in Figure 4 and fit to a "Michaelis-Menten-like" enzyme kinetics model to derive k_{react} and $K_{M(sto)}$.

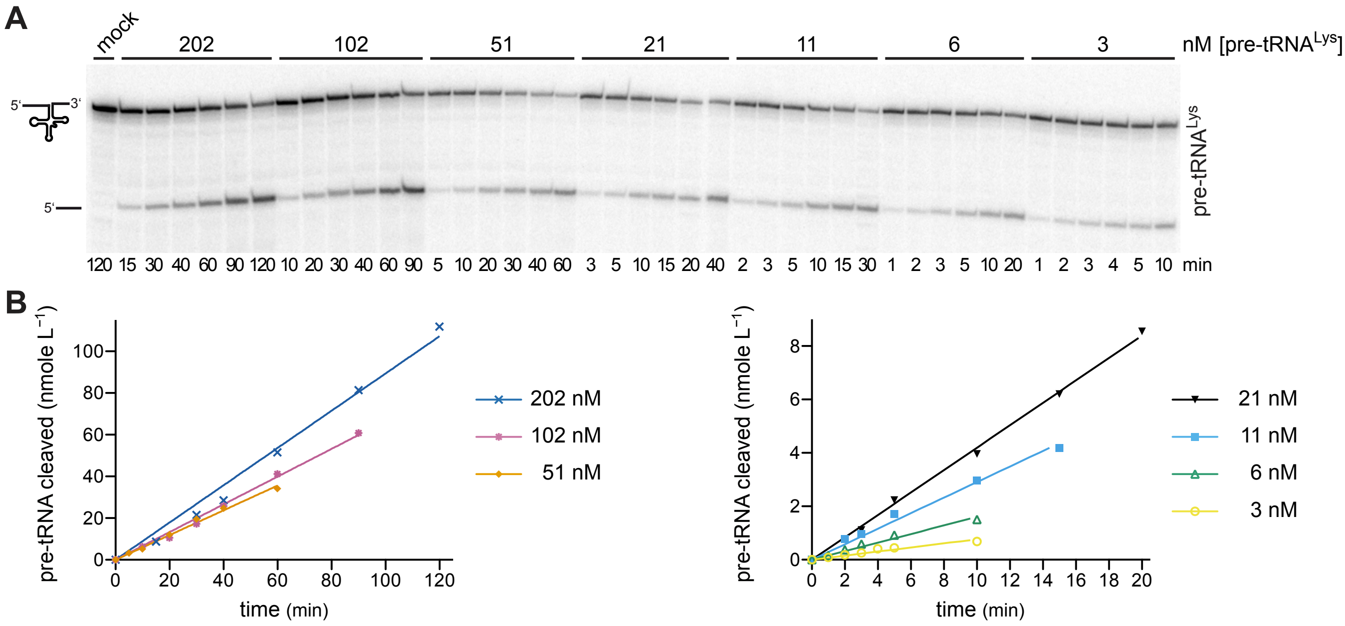

Supplementary Figure S5. Multiple-turnover kinetic analysis of cleavage by the mtRNase P holoenzyme (PRORP plus TRMT10C-SDR5C1); a representative example using mitochondrial pre-tRNA^{Lys} as substrate. (**A**) Reactions with the indicated concentrations of pre-tRNA^{Lys} were carried out as described in the Materials and Methods section of the main text (at 4.5 mM Mg^{2+}). Aliquots were withdrawn and the reaction stopped at the indicated time points. Samples were analyzed by gel electrophoresis and phosphorimaging. (**B**) Radioactive bands representing pre-tRNA and 5′-cleavage product were quantified to determine the cleaved substrate fraction, which was converted to nmole L^{-1} cleaved. Data from the initial linear phase of the reaction were fit by linear regression analysis to approximate the initial velocity (*v*) at each PRORP concentration; for better graphical representation, data are divided into two differently scaled graphs here. Initial velocities (*v*) were plotted in graphs as shown in Figure 7 and fit to a Michaelis-Menten enzyme kinetics model to derive k_{cat} and K_M.

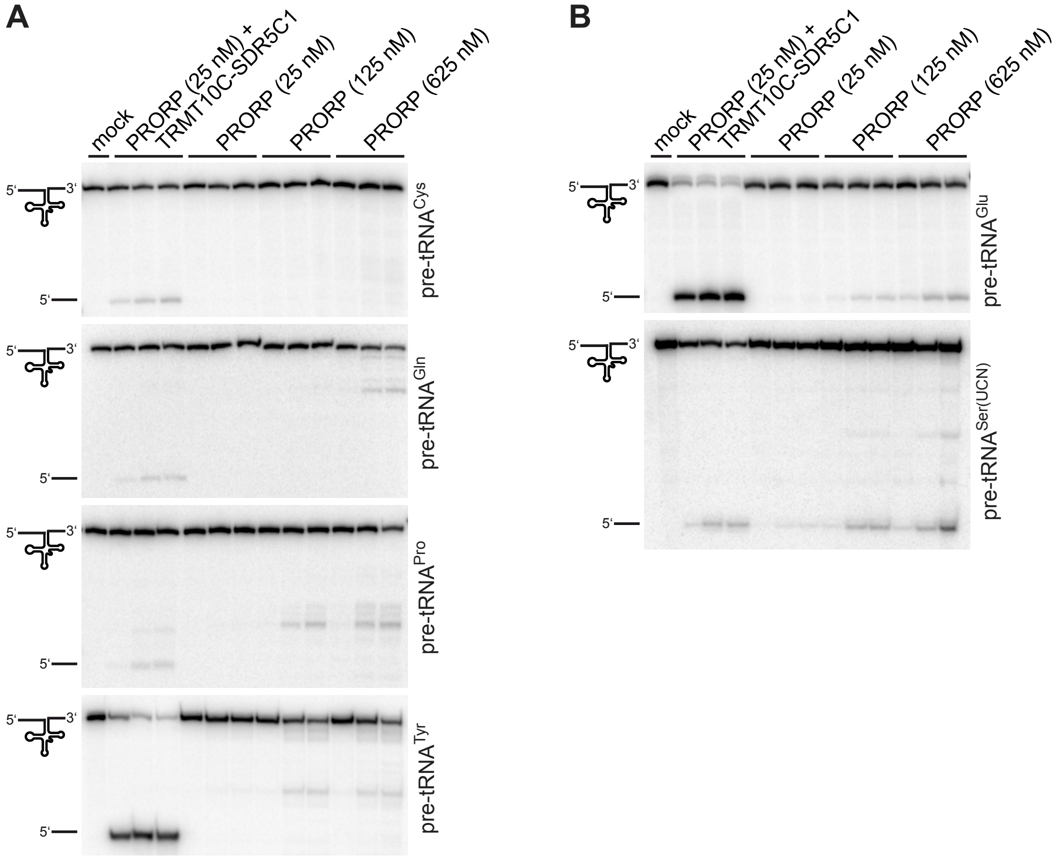

Supplementary Figure S6. RNase P activity of PRORP without TRMT10C-SDR5C1 (analysis of more mitochondrial pre-tRNAs). The RNase P activity of PRORP was tested with six additional human mitochondrial pre-tRNAs. Aliquots were withdrawn from the reactions after 3, 30, and 60 min; cleavage products were separated by gel electrophoresis and visualized by phosphorimaging. The final concentration of the TRMT10C-SDR5C1 complex was 250 nM. No enzyme was added to the “mock” reaction, which was incubated for 60 min. Due to 5’-end labelling, only the full-length pre-tRNA and the released 5’ leader are visible. (**A**) Examples of human mitochondrial pre-tRNAs that required both, PRORP and the TRMT10C-SDR5C1 complex for 5’-end processing. (**B**) Examples of human mitochondrial pre-tRNAs on which PRORP alone showed substantial RNase P activity *in vitro*.

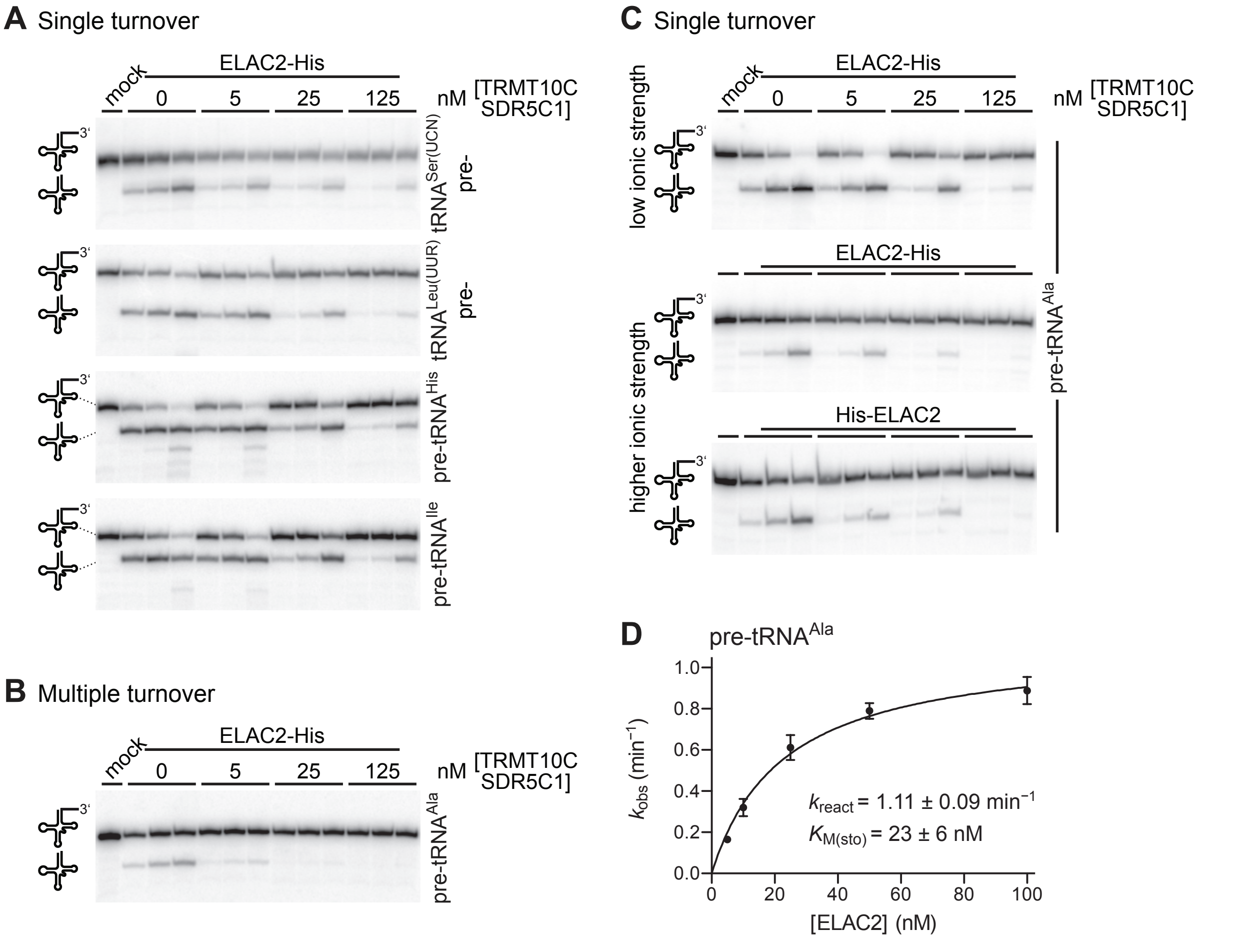

Supplementary Figure S7. Effect of TRMT10C-SDR5C1 on the activity of RNase Z (**A**) The effect of the TRMT10C-SDR5C1 complex on the RNase Z activity of ELAC2 was analyzed (under single-turnover conditions) for four additional human mitochondrial pre-tRNAs as described in Figure 3A. Mitochondrial pre-tRNAs with mature 5' ends (Supplementary Figure S1B) were supplemented with the indicated concentrations of TRMT10C-SDR5C1 and tested for 3' processing by ELAC2-His (25 nM); aliquots were withdrawn from the reactions after 1, 5, and 60 min, and cleavage products separated by gel electrophoresis and visualized by phosphorimaging. (**B**) The effect of the TRMT10C-SDR5C1 on the RNase Z activity of ELAC2 was analyzed under multiple-turnover conditions. Pre-tRNA^{Ala} (25 nM) was supplemented with the indicated concentrations of TRMT10C-SDR5C1, and subjected to cleavage by ELAC2-His (1 nM); aliquots were withdrawn after 30, 60, and 120 min, and analyzed as described above. (**C**) The effect of the TRMT10C-SDR5C1 complex on the RNase Z activity of ELAC2 was analyzed (under single-turnover conditions) with C- and N-terminally tagged ELAC2 in low ionic strength (70 mM) and higher ionic strength buffer (150 mM), the latter corresponding to the buffer previously used by others (ref. 53). Pre-tRNA^{Ala} was supplemented with the indicated concentrations of TRMT10C-SDR5C1, and analyzed for cleavage by the indicated variant of recombinant ELAC2 (25 nM); aliquots were withdrawn at 1, 5, and 60 min, and analyzed as described above. (**D**) Single-turnover kinetic analyses of the RNase Z activity of ELAC2. First-order rate constants of cleavage (k_{obs}) were plotted against PRORP concentration. Data points are the mean ± SEM of at least 5 replicates. Derived kinetic constants k_{react} and $K_{M(sto)}$ (best-fit values ± curve-fit standard error) are inserted into the graph.

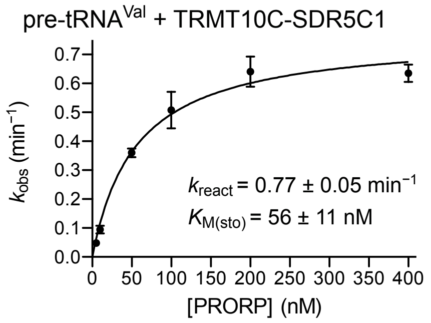

Supplementary Figure S8. Single-turnover kinetics of pre-tRNA^{Val} cleavage by mtRNase P. Single-turnover kinetic analysis was performed with PRORP in the presence of an excess of TRMT10C-SDR5C1 complex (mtRNase P holoenzyme; 4.5 mM Mg^{2+}). Pseudo first-order rate constants of cleavage (k_{obs}) were plotted against the concentration of PRORP. Data points are the mean ± SEM of at least 5 replicates. Derived kinetic constants k_{react} and $K_{M(sto)}$ (best-fit values ± curve-fit standard error) are inserted into the graph.

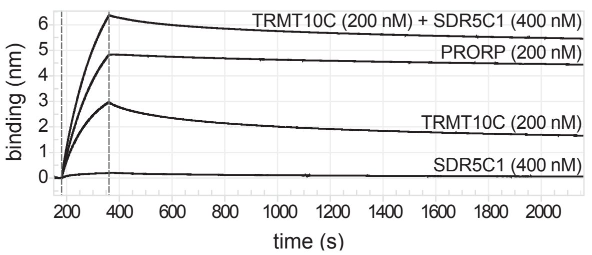

Supplementary Figure S9. Binding of the subunits of mtRNase P to pre-tRNA^{Lys}. Fitting of representative bio layer interferometry signals upon binding of the subunits of mtRNase P to pre-tRNA^{Lys}. Association and dissociation phases are displayed. Each binding curve is labelled with the protein ligand(s) and its/their concentration.

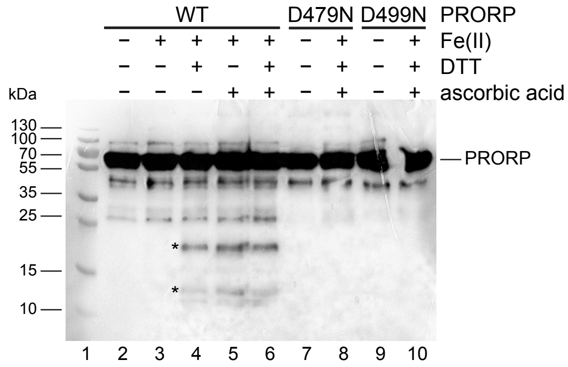

Supplementary Figure S10. Iron-mediated hydroxyl radical cleavage of PRORP: confirmation of C-terminal cleavage products. PRORP was subjected to iron-mediated hydroxyl radical cleavage exactly as described in Figure 5A. After SDS-PAGE, the gel was blotted onto PVDF membrane and probed with an antibody against the C-terminal His-tag of PRORP. The two C-terminal fragments of PRORP (lanes 4, 5, and 6) are indicated by asterisks. The molecular weight of selected size markers (lane 1) is indicated to the left. The position of full-length PRORP is indicated to the right.
